# Genome-wide investigation and expression profiling of polyphenol oxidase (PPO) family genes uncover likely functions in organ development and stress responses in *Populus trichocarpa*

**DOI:** 10.1186/s12864-021-08028-9

**Published:** 2021-10-08

**Authors:** Fang He, Yu-Jie Shi, Qian Zhao, Kuang-Ji Zhao, Xing-Lei Cui, Liang-Hua Chen, Han-Bo Yang, Fan Zhang, Jia-Xuan Mi, Jin-Liang Huang, Xue-Qin Wan

**Affiliations:** Sichuan Province Key Laboratory of Ecological Forestry Engineering on the Upper Reaches of the Yangtze River, College of Forestry, Sichuan Agricultural University, Chengdu, 611130 China

**Keywords:** Genome-wide, *Populus*, *PPO* gene family, Expression pattern, Stress

## Abstract

**Background:**

Trees such as *Populus* are planted extensively for reforestation and afforestation. However, their successful establishment greatly depends upon ambient environmental conditions and their relative resistance to abiotic and biotic stresses. Polyphenol oxidase (PPO) is a ubiquitous metalloproteinase in plants, which plays crucial roles in mediating plant resistance against biotic and abiotic stresses. Although the whole genome sequence of *Populus trichocarpa* has long been published, little is known about the *PPO* genes in *Populus*, especially those related to drought stress, mechanical damage, and insect feeding. Additionally, there is a paucity of information regarding hormonal responses at the whole genome level.

**Results:**

A genome-wide analysis of the poplar PPO family was performed in the present study, and 18 *PtrPPO* genes were identified. Bioinformatics and qRT-PCR were then used to analyze the gene structure, phylogeny, chromosomal localization, gene replication, *cis*-elements, and expression patterns of *PtrPPOs*. Sequence analysis revealed that two-thirds of the *PtrPPO* genes lacked intronic sequences. Phylogenetic analysis showed that all *PPO* genes were categorized into 11 groups, and woody plants harbored many *PPO* genes. Eighteen *PtrPPO* genes were disproportionally localized on 19 chromosomes, and 3 pairs of segmented replication genes and 4 tandem repeat genomes were detected in poplars. *Cis*-acting element analysis identified numerous growth and developmental elements, secondary metabolism processes, and stress-related elements in the promoters of different PPO members. Furthermore, *PtrPPO* genes were expressed preferentially in the tissues and fruits of young plants. In addition, the expression of some *PtrPPOs* could be significantly induced by polyethylene glycol, abscisic acid, and methyl jasmonate, thereby revealing their potential role in regulating the stress response. Currently, we identified potential upstream TFs of *PtrPPOs* using bioinformatics.

**Conclusions:**

Comprehensive analysis is helpful for selecting candidate *PPO* genes for follow-up studies on biological function, and progress in understanding the molecular genetic basis of stress resistance in forest trees might lead to the development of genetic resources.

**Supplementary Information:**

The online version contains supplementary material available at 10.1186/s12864-021-08028-9.

## Background

Aggravated global warming induces more frequent and severe the local stress in whole ecosystems [1, 2]. Plants have evolved complex mechanisms to defend themselves against various biotic and abiotic stresses [3, 4]. For example, plants frequently encounter insect pests and have evolved an efficient immune response that mainly depends on producing specific metabolites and defense proteins [5, 6]. At the same time, plants competently transform their morphological structure, synthesize favorable metabolites, and evolve complex molecular mechanisms to deal with abiotic stresses [7–9], such as drought, cold, heat, and salt stress.

Polyphenol oxidase (PPO) is a widely distributed metalloproteinase that primarily exists in plants, fungi, and insects [10]. It can not only catalyze the oxidation of catechol to quinones but also acts on monophenol monooxygenase substrates. In a broad sense, PPO can be classified into three categories, i.e., tyrosinase (EC.1.14.18.1), catechol oxidase (EC.1.10.3.2), and laccase (EC.1.10.3.1) [11]. Of these three types of PPO, catecholase is mainly distributed in plants, while laccase and tyrosinase are distributed in microorganisms [12]. The function of PPO in plants is largely determined by three conserved domains i.e., KFDV, tyrosinase, and DWL [13].

PPO proteins predominantly exist in terrestrial plants, such as apple, litchi, spinach, potato, legume, tea, mulberry leaf, tobacco, and grapevine, as well as in fungi and some bacteria [11]. Under normal circumstances, PPO is inactive and tightly binds to the inner capsule membrane; activated PPO plays an important role after tissue damage. In addition, several PPO genes have been identified in *Musa acuminata*, *Malus domestica, Oryza sativa*, *Sorghum bicolor*, *Solanum tuberosum, Ananas comosus,* and *Cucumis sativus* [11, 14–19]. However, there are considerable differences in the distribution and function of PPO proteins in different plants. The location of PPO varies with plant species and maturity level; nevertheless, in most plant leaves, PPO is primarily distributed in chloroplasts [13], while almost all sub-cells of potato tubers contain PPO [12]. Polyphenol oxidase in tea exists in dissociative and combinative states; the former is chiefly found in the cytoplasm, while the latter is mainly found in chloroplasts, mitochondria, and other organelles [11, 20]. Majority of studies have revealed that PPO activity and polyphenol content in tea shoots significantly affect the quality of red tea [21].

Extensive literature mining has revealed that the expression of PPO genes in plants is closely related to stress and response to insect and mechanical damage, diseases, and microorganism invasion [11, 22]. Additionally, the *PPO* gene family exhibits a heightened response to methyl jasmonate (MeJA) in *Salvia miltiorrhiza* and *Nicotiana tabacum L* [23, 24]. Moreover, overexpression of the *PPO* gene in tomato can enhance plant resistance to insect pests, including *Spodoptera litura*, *Helicoverpa armigera*, and *Spodoptera exigua* [25, 26]. Similarly, the overexpression of *PPO* genes in poplar trees can result in inhibition of the growth of *Malacosoma disstria* [27]. Furthermore, upregulation of *PPO* expression in *Lycopersicon esculentum, Juglans regia, Taraxacum officinale,* and *Fragaria ananassa* enhances plant resistance to fungi [11, 25]. Some key enzymes in the phenolic metabolic system play an essential role in mediating the resistance of plants to pathogenic microorganisms. For example, the pathogen-related protein PPO can catalyze the formation of lignin and quinones, thus promoting the formation of defensive barriers and protecting cells from invasion by strengthening the cell structure [28].

*Populus* has many advantages in basic research as a typical model woody plant with relatively upper ecological, economic, and cultural significance [29–31]. Stress severely restricts poplar cultivation in plantations. There are few functional studies on the role of the PPO protein family in biotic and abiotic stress in poplar trees. Here, we identified 18 putative PPO proteins in the *Populus trichocarpa* genome. Comparative genomics, transcriptomics, and RT-qPCR were used to comprehensively analyze the PPO protein family in poplar to provide a theoretical basis for studies on the characteristics and functions of PPOs in poplar development and stress response.

## Results

### Identification and analysis of poplar *PPOs*

Eighteen putative *PPO* genes were identified from the published *P. trichocarpa* reference genome sequence and were successively designated *PtrPPO1* to *PtrPPO18* according to their location on the genome (Figure S1). In order to clearly understand the characteristics of the *PPO* family in poplar, we analyzed the gene length, transcriptional sequence length, CDS (coding sequence) length, the position of the conserved domain, amino acid (AA) length, protein molecular weight (MW), grand average of hydropathicity (GRAVY), and isoelectric point (PI) of the proteins encoded by these genes (Table 1).

**Table 1:** Summary of *Populus PPO* genes

Name	Gene model ID	gDNA	Transcript	CDS	Domains	AA	MW	GRAVY	PI
PtrPPO1	Potri.001G387900	1921	1921	1746	159-367/377-428/453-578	581	64.913	-0.453	6.00
PtrPPO2	Potri.001G388000	662	579	579	5-44/47-97/110-189	192	22.200	-0.821	5.74
PtrPPO3	Potri.001G388100	2552	1584	1566	91-307/317-368/393-518	521	58.651	-0.413	5.35
PtrPPO4	Potri.001G388200	1927	1927	1746	159-367/377-428/453-578	581	64.757	-0.436	5.21
PtrPPO5	Potri.001G388300	2009	1326	1326	74-227/237-287/313-438	441	49.561	-0.527	5.28
PtrPPO6	Potri.001G388400	1752	1752	1746	159-367/377-428/453-578	581	64.999	-0.449	5.96
PtrPPO7	Potri.001G388600	1805	1805	1746	159-367/377-428/453-578	581	64.667	-0.424	5.68
PtrPPO8	Potri.001G388800	1940	1940	1746	159-367/377-428/453-578	581	65.008	-0.449	6.14
PtrPPO9	Potri.001G388900	2149	2149	1821	193-400/407-455/482-603	606	68.678	-0.450	6.53
PtrPPO10	Potri.004G038500	1346	1346	744	118-244	247	27.230	-0.515	6.93
PtrPPO11	Potri.004G156500	2303	2140	1764	140-357/363-413/453-585	587	67.459	-0.337	6.36
PtrPPO12	Potri.011G047200	1058	1058	732	115-240	243	26.419	-0.419	6.21
PtrPPO13	Potri.011G047300	2135	2135	1773	160-368/374-425/460-587	590	66.288	-0.536	6.55
PtrPPO14	Potri.011G108200	3295	1713	1713	184-348/358-408/430-522	570	64.747	-0.516	7.53
PtrPPO15	Potri.011G108300	2013	2013	1692	145-351/361-411/433-560	563	64.066	-0.514	5.96
PtrPPO16	Potri.T061900	1921	1914	1746	159-367/377-428/453-578	581	65.077	-0.455	6.23
PtrPPO17	Potri.T062100	1889	1688	1638	159-331/341-392/417-542	545	60.654	-0.420	5.86
PtrPPO18	Potri.T062200	1947	1947	1746	159-367/377-428/453-578	581	64.889	-0.442	6.30

Among the 18 PtrPPO proteins, PtrPPO2 was the shortest with 192 amino acids, whereas PtrPPO9 was the longest (606 AA). The MW of PtrPPO proteins was 22.200 to 68.678 kDa, the GRAVY of the proteins was − 0.821 (PtrPPO2) to − 0.337 (PtrPPO11), and PI was 5.21 (PtrPPO4) to 7.53 (PtrPPO14). Generally, identifying the molecular characteristics of PPOs will be helpful in studying their specific biological functions.

### Evolutionary and phylogenetic analysis of the PPO family

**Fig. 1:**
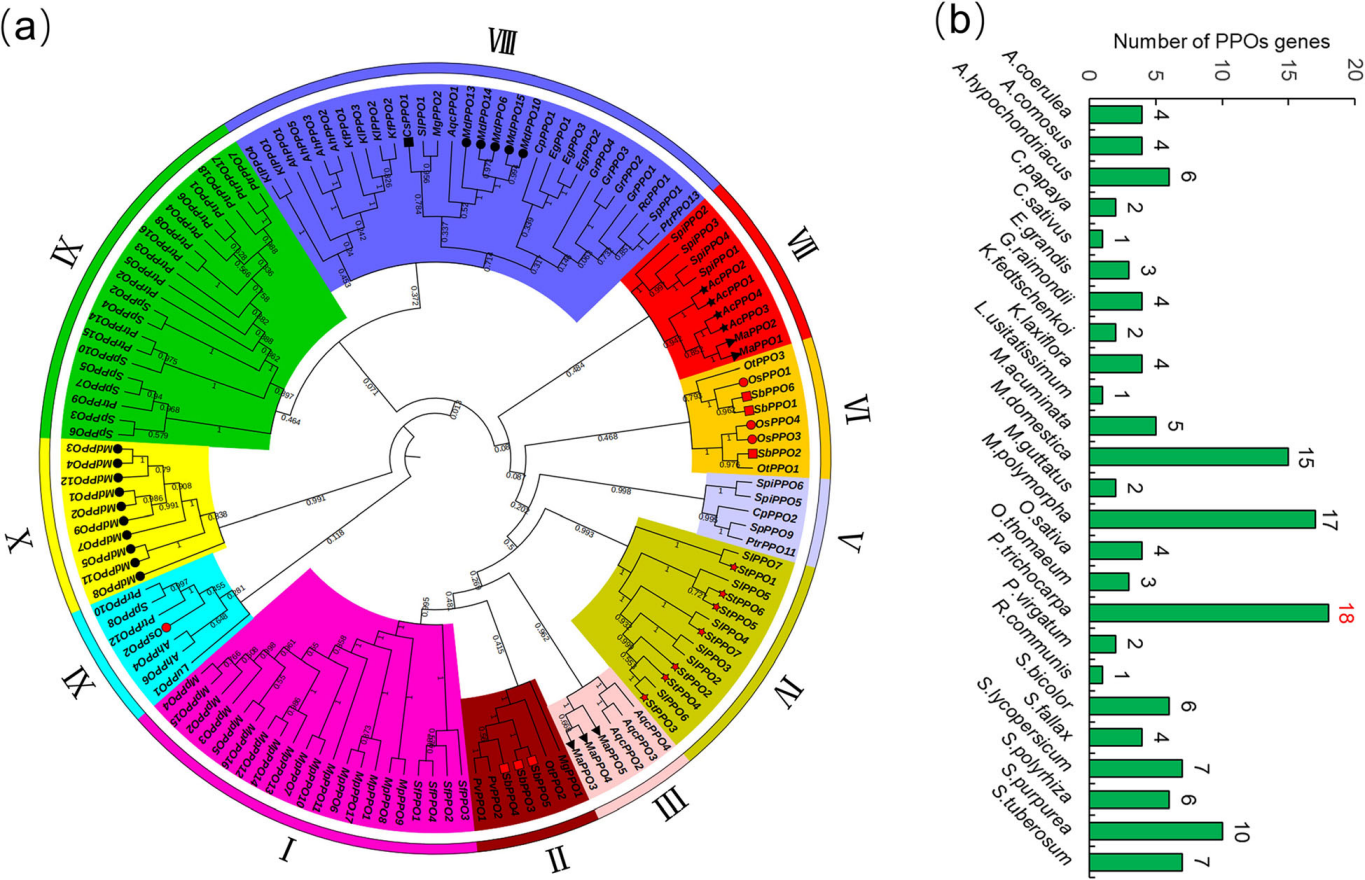
Evolutionary and phylogenetic analysis of the PPO family in diverse plant species. **a** The phylogenetic tree of PPO proteins from *Aquilegia coerulea (AqcPPO), Ananas comosus (AcPPO), Amaranthus hypochondriacus (AhPPO), Carica papaya (CpPPO), Cucumis sativus (CsPPO), Eucalyptus grandis (EgPPO), Gossypium raimondii (GrPPO), Kalanchoe fedtschenkoi (KfPPO), Kalanchoe laxiflora (KlPPO), Linum usitatissimum (LuPPO), Musa acuminata (MaPPO), Malus domestica (MdPPO), Mimulus guttatus (MgPPO), Marchantia polymorpha (MpPPO), Oryza sativa (OsPPO), Oropetium thomaeum (OtPPO), Populus trichocarpa (PtrPPO), Panicum virgatum (PvPPO), Ricinus communis (RcPPO), Sorghum bicolor (SbPPO), Sphagnum fallax (SfPPO), Solanum lycopersicum (SlPPO), Spirodela polyrhiza (SpiPPO), Salix purpurea (SpPPO),* and *Solanum tuberosum (StPPO)*. The shape and color in front of the node represent the identified *PPOs*, in which the black circle represents *MdPPOs*, the black rectangle represents *CsPPOs*, the black pentagram represents *AcPPOs*, the black triangle represents *MaPPOs*, the red circle represents *OsPPOs*, the red rectangle represents *SbPPOs*, and the red pentagram represents *StPPO*s. **b** Comparisons of PPO protein numbers across 25 plant species. The accession numbers and gene names are all shown in Table S2

To better understand the evolution and differentiation of PPO family proteins among species, 138 PPO amino acid sequences from 25 species were used to construct an unrooted tree (Fig. 1a). These sequences were then divided into 11 groups (I–XI), among which groups VIII and IX contained the most members, including 51 members accounting for 36.96% of the deduced PPO protein. PtrPPO protein was found in groups V, VIII, IX, and XI. Simultaneously, 14 PPO proteins in poplar were concentrated in group IX and clustered in the same branch as 7 PPO proteins in *Salix purpurea*.

To determine the origin and evolution of *PPO* genes, we searched for *PPO* genes in 25 species of lower aquatic plants and higher terrestrial plants (Fig. 1b). Interestingly, this gene was absent in *Arabidopsis*, but four *PPO* genes were found in monocotyledonous *O. sativa*. It is worth noting that *PPO* genes are widely distributed in woody plants, especially in *P. trichocarpa* (18), *M. domestica* (15), and *S. purpurea* (10). However, *Marchantia polymorpha* (common liverwort, an herbaceous plant) contains 17 *PPO* genes. In conclusion, the distribution of *PPO* genes in woody plants was much greater than that in herbaceous plants, indicating that considerable differentiation and doubling of *PPO* genes might have occurred during the evolution of perennial woody plants.

### Determination of gene structure, conserved domains, and motifs

**Fig. 2:**
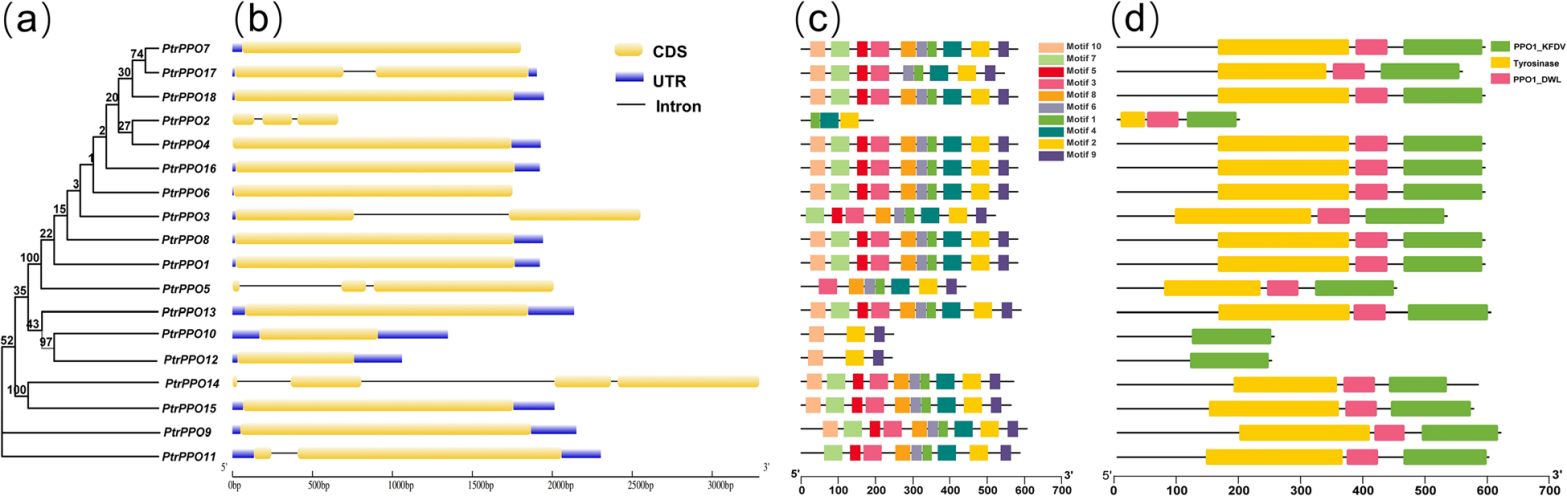
Phylogenetic, gene structure, conserved protein structure, and motif analyses of *PtrPPOs*. **a** Phylogenetic tree constructed using full-length amino acid sequences from 18 *PtrPPO*s. **b** Exon-intron structure analysis of *PtrPPO* genes. Blue boxes represent UTRs; yellow boxes indicate exons; black lines represent introns. **c** Motif distribution of PtrPPO members. **d** conserved protein domain analysis of PtrPPOs. The length of each pattern is displayed proportionally

To explore the evolutionary relationship between different members of the *PPO* gene family more clearly, a phylogenetic tree was constructed using 18 PPO proteins from *Populus* (Fig. 2a), and their gene structure, conserved protein motifs, and conserved structure were assessed. Most *PtrPPO* genes (approximately two-thirds of the genes) had no introns, while the other six genes (*PtrPPO2/3/5/11/14/17*) had at most three introns. Surprisingly, none of the genes with two and three introns had a UTR (Fig. 2b). For example, *PtrPPO7/8/1* genes lacked an intron, but they all had UTRs. However, *PtrPPO2/5/14* contained at least two introns but no UTR.

Generally, genes with high similarity in amino acid sequences in the same family may have similar biological functions. We identified three conserved domains (PPO1-KFDV, tyrosinase, and PPO1_DWL) in the PPO protein sequence using CDD and SMART software (Fig. 2d and Table S3). All PPO proteins, except for PtrPPO10/12, had these three structures, which may endow them with similar biological functions. Meanwhile, 10 conserved motifs were identified in the PtrPPOs protein sequence using MEME (Fig. 2c). Amino acid sequence encode, and SeqLogo of the Motif are displayed in Table S4. Among them, two motifs (2 and 9) were related to the C-terminal PPO-KFDV domain, motif 4 was related to the intermediate PPO_DWL domain, and five motifs (1, 6, 8, 3, and 5) were related to the N-terminal tyrosinase domain. Moreover, we found two novel motifs (motifs 7 and 10) at the N-terminal of most PtrPPO protein sequences.

### Analysis of chromosomal location and gene duplication

**Fig. 3:**
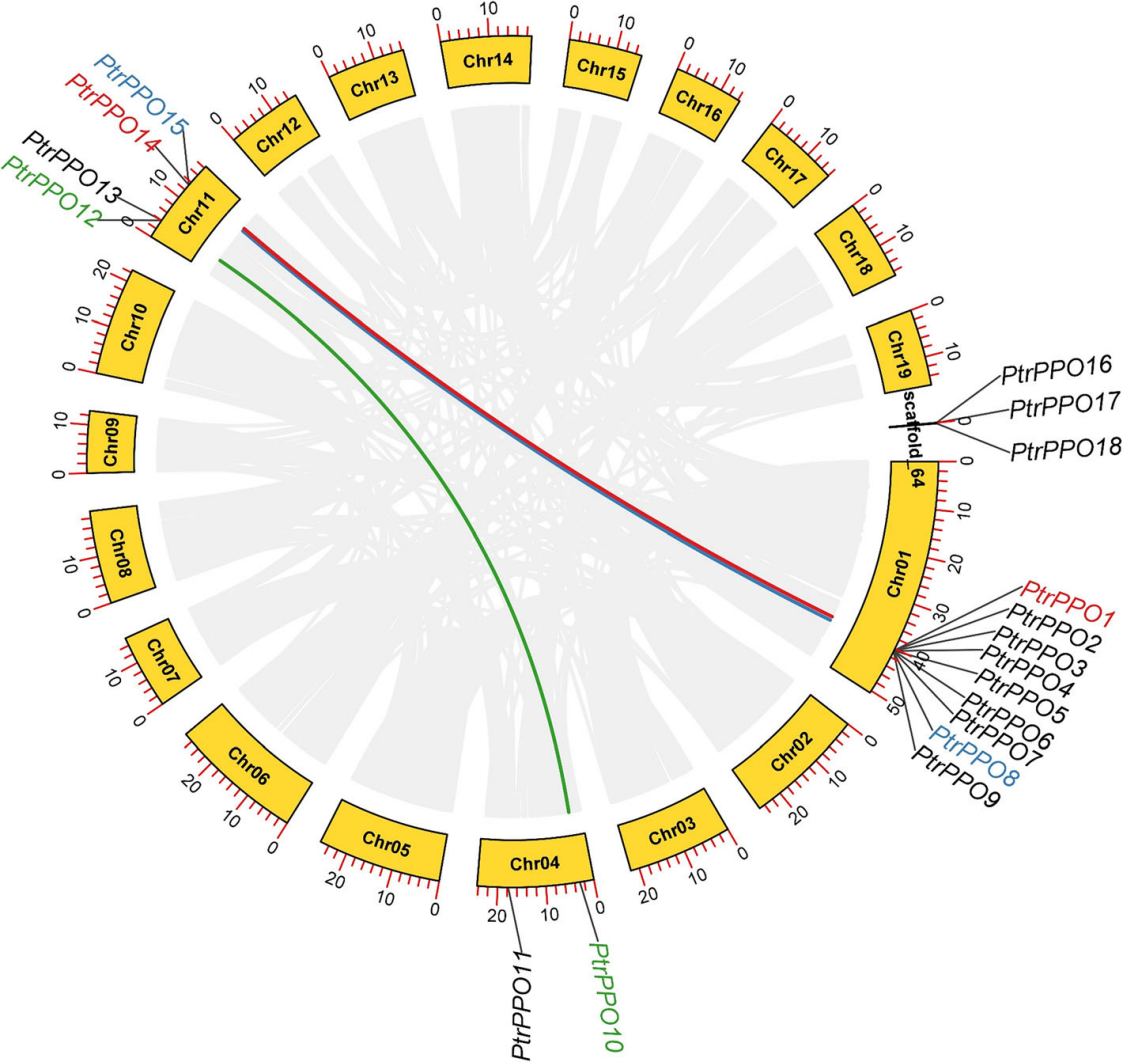
Schematic diagram depicting PPO gene distribution and Inter-chromosomal relationships in *Populus* chromosomes. The gray lines represent all synteny blocks in the chromosome of *Populus trichocarpa*. Red, blue, and green lines indicate duplicated *PPO* gene pairs in *Populus trichocarpa.* Chromosome number is shown in the middle of the arc square. The length of each arc corresponds to the length of the chromosome (Mb)

To further understand the evolution and differentiation of *PPO* family genes, we analyzed the chromosomal distribution, synteny, and evolution of the 18 *PPO* genes in *Populus*. The *PPO* genes were primarily distributed on chromosomes 1, 4, 11, and scaffold_64 (Fig. 3 and Figure S1). Moreover, half of the *PPO* genes in poplar were distributed on chromosome 1, while there were, at least, two *PPO* genes on the other chromosomes. It has been reported that the chromosome region within 200 kb containing two or more genes is defined as a tandem replication event [32]. The 18 genes (*PtrPPO1/2/3/4/5/6/7/8/9, PtrPPO12/13, PtrPPO14/15, PtrPPO16/17/18*) formed four tandem repeat regions distributed on chromosomes 1, 11, and scaffold_64 (Fig. 3). The high sequence similarity between repetitive gene pairs indicates that they are likely involved in regulating similar biological processes. Furthermore, nine *PPO* genes formed a complex tandem repeat on chromosome 1, indicating a hot spot for *PPO* gene distribution. We also found that six genes (*PtrPPO1/14, PtrPPO8/15, PtrPPO10/12*) formed three segmental duplication events using the MCScanX method. These results suggest that *PtrPPOs* might be produced by gene replication, while tandem duplication and repetitive fragment events collectively catalyze the evolution of *PPO* genes in poplar.

**Fig. 4:**
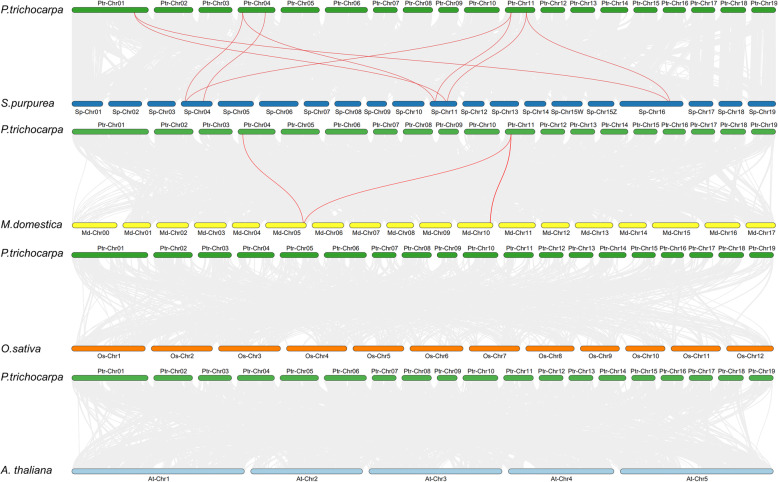
Collinearity analysis of *PPO* genes in poplar and four typical plant species. The gray lines in the background represent collinear blocks between *Populus* and another species’ genomes, while the red lines highlight the syntenic PPO gene pairs. *P.trichocarpa*, *Populus trichocarpa*; *S.purpurea, Salix purpurea; M.domestica, Malus domestica; O. sativa, Oryza sativa*

We constructed comparative syntenic maps between poplar and four other species to analyze PPO gene evolution in woody plants further. Syntenic maps revealed that nine pairs of homologous genes were found between *P. trichocarpa* and *S. purpurea*, and three pairs of homologous genes were found between *P. trichocarpa* and *M. domestica* (Fig. 4 and Table S5). Some *PtrPPO* genes (*PtrPPO10* and *PtrPPO12*) were discovered to be connected with at least two synonym gene pairs (especially between poplar and willow, and between poplar and apple *PPO* genes), suggesting that these genes may play essential roles in the evolutionary process of the *PPO* gene family. However, the findings of previous studies and those of the present study revealed that there was no synteny in *Populus* vs. *O. sativa* and *Populus* vs. *A. thaliana* [11], suggesting that multiple *PPO* genes may be formed during the differentiation of woody plants.

### *PtrPPO cis*-element analysis

**Fig. 5:**
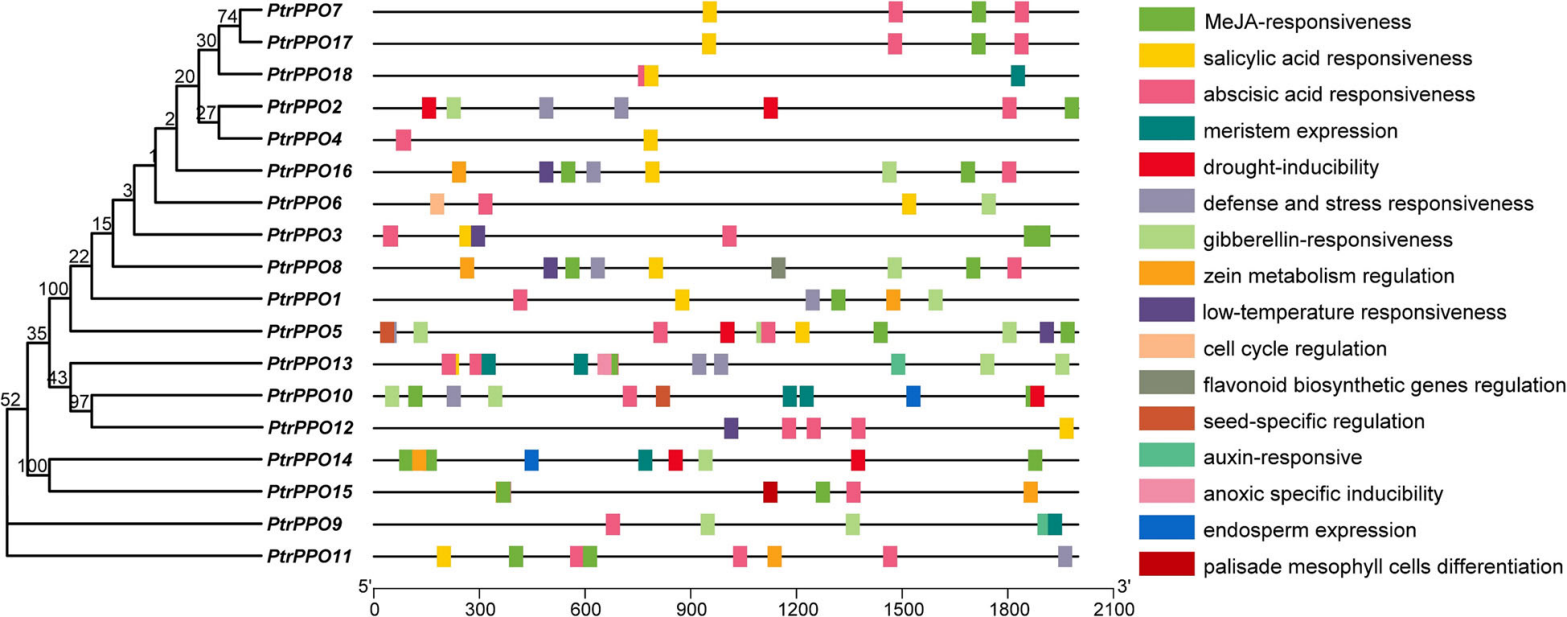
*Cis*-element analysis of the *PtrPPOs*. The relative locations of stress and growth-related *cis*-elements in the *PtrPPO* promoter region. Different colors represent different *cis*-acting elements, and their position corresponds to the corresponding position of the promoter

To determine the expression pattern of *PtrPPOs*, the TSS promoter-upstream region (~ 2000 bp) sequences were extracted from the genomic DNA sequence of *P. trichocarpa*. The *cis*-elements of the *PtrPPOs* promoter were analyzed using the PlantCARE database (Fig. 5). The specific functions of these motifs (*cis*-elements) are annotated in Table S6. A series of *cis*-acting elements involved in all aspects of poplar development, including palisade mesophyll cell differentiation, seed-specific regulation, cell cycle regulation, endosperm expression, meristem expression, and gibberellin-responsive and auxin-responsive elements, were identified, indicating the crucial role of *PtrPPOs* in poplar development. For example, the promoter regions of many *PPO* genes were identified to contain gibberellin-responsive elements, and the *PtrPPO5* promoter contained three gibberellin-responsive elements, indicating that this gene is likely involved in gibberellin-signal transduction (Table S7). Moreover, the promoter region of *PtrPPO*10 contains elements of cell cycle regulation, meristem expression, and endosperm expression, indicating that this gene may be involved in the regulation of poplar growth and development.

Furthermore, *PtrPPO* promoter regions were associated with secondary metabolism processes, including zein metabolism regulation and flavonoid biosynthetic gene regulation. *PtrPPO*8 contained elements related to the aforementioned processes, suggesting that this gene might be involved in secondary metabolism in *Populus*. Besides, some stress-related elements were present in the promoter region, including MeJA responsiveness, salicylic acid responsiveness, abscisic acid responsiveness, drought inducibility, defense and stress responsiveness, anoxic specific inducibility elements, and low-temperature responsiveness, which implied that *PtrPPOs* might also respond to stress. Except for *PtrPPO*4/6/9/12/18, all other *PPO* genes contained MeJA-responsive elements (Table S7), indicating that most *PPO* genes were involved in MeJA signal transduction. Moreover, *PtrPPO13* harbored multiple elements associated with stress, including two MeJA-responsive elements, one salicylic-acid-responsive element, four abscisic-acid-responsive elements, and two defense and stress responsive elements (Table S7), indicating that *PtrPPO13* may respond to both biological and abiotic stresses.

### RNA-seq analysis of *Populus PPO* genes

**Fig. 6:**
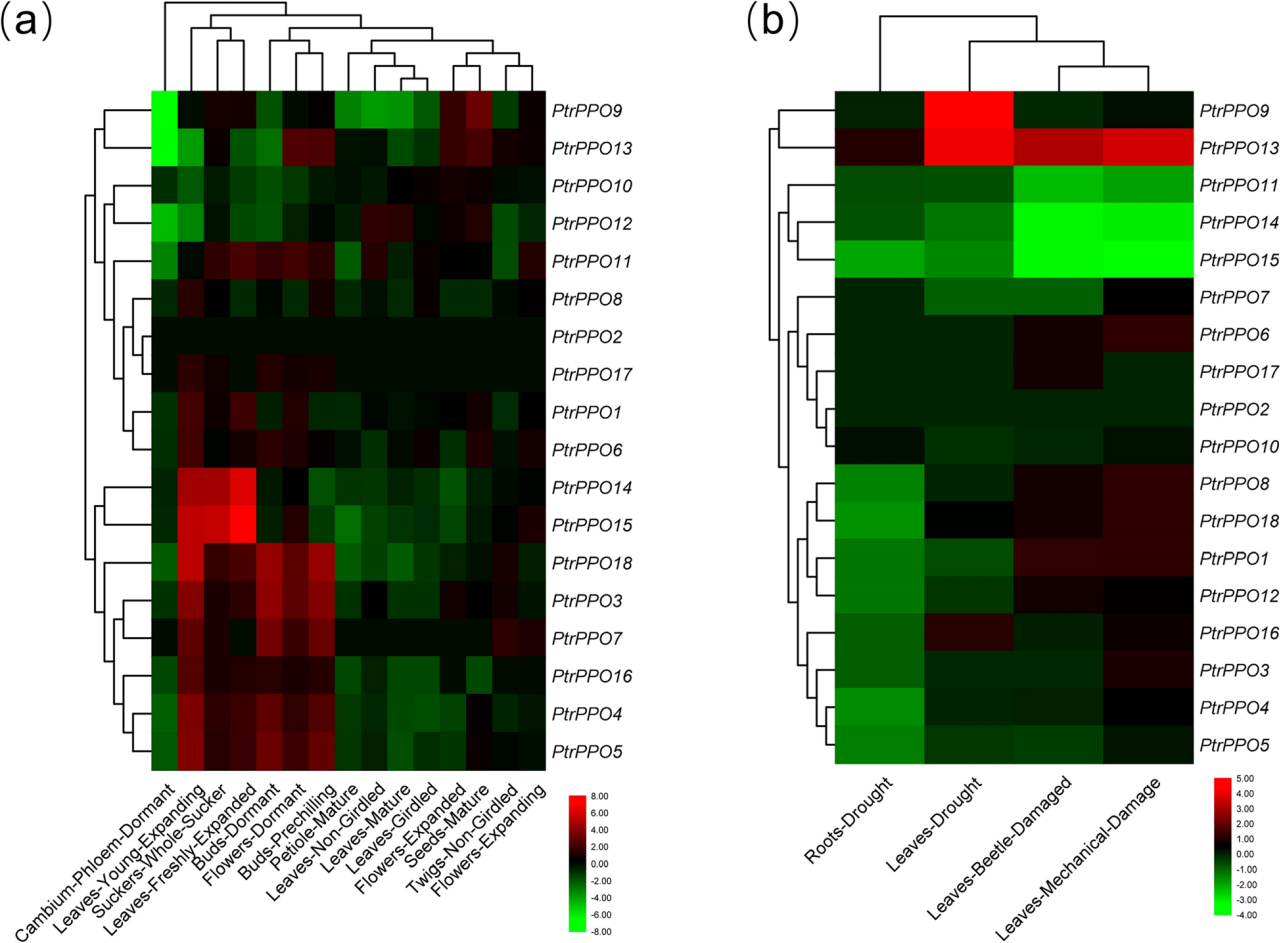
Expression profile of *PPO* genes under developmental and stress conditions. **a** Heat maps showing the expression levels of 18 *PtrPPO* genes in different tissues at different developmental stages based on transcriptome data. **b** Heat maps depicting the expression levels of 18 *PtrPPO* genes following drought-, beetle-, and mechanical damage. The color bar represents the range of maximum and minimum values of relative expression in the heat map

To explore the role of *PPO* genes in poplar growth and development, we analyzed the tissue expression patterns of 18 *PtrPPOs* in the transcriptomic dataset. First, we found that *PtrPPO*2 was not detected in all tissues and that it might be a pseudogene or might have a specific spatiotemporal expression pattern (not included in the library). Next, we constructed a dual clustering heat map (sample and gene) to investigate the expression profile of these genes in 15 *Populus* tissues (Fig. 6a and Table S8). Interestingly, some genes were preferentially expressed in specific plant tissues. Eight genes were highly expressed in young expanding leaves (*PtrPPO14/15/18/3/7/16/4/5*), two genes in whole suckers and freshly expanding leaves (*PtrPPO14/15*), five genes in dormant and prechilled buds (*PtrPPO18/3/7/4/5*), and one gene in mature seeds (*PtrPPO9*). It was further revealed that most of these genes were found in young plant tissues or seeds. For example, *PtrPPO18* was expressed at high levels in young tissues but at low levels in mature tissues (such as mature leaves and petioles).

To further understand the role of *PPO* genes in the response of poplar plants to stress, we mapped the expression configurations of the 18 poplar *PPO* genes following exposure to drought and beetle- and mechanical-damage using the uploaded transcriptome dataset (Fig. 6b and Table S9). The heat map revealed that the expression of *PtrPPO9* was significantly induced in leaves in response to drought stress, and all three treatments significantly induced the expression of *PtrPPO13*. However, the expression of *PtrPPO11/14/15* was significantly inhibited under conditions of beetle- and mechanical-damage-induced stress. These results will be helpful for future research on gene function.

### Expression profile of *Populus PPO* genes in different plant tissues and treatments

**Fig. 7:**
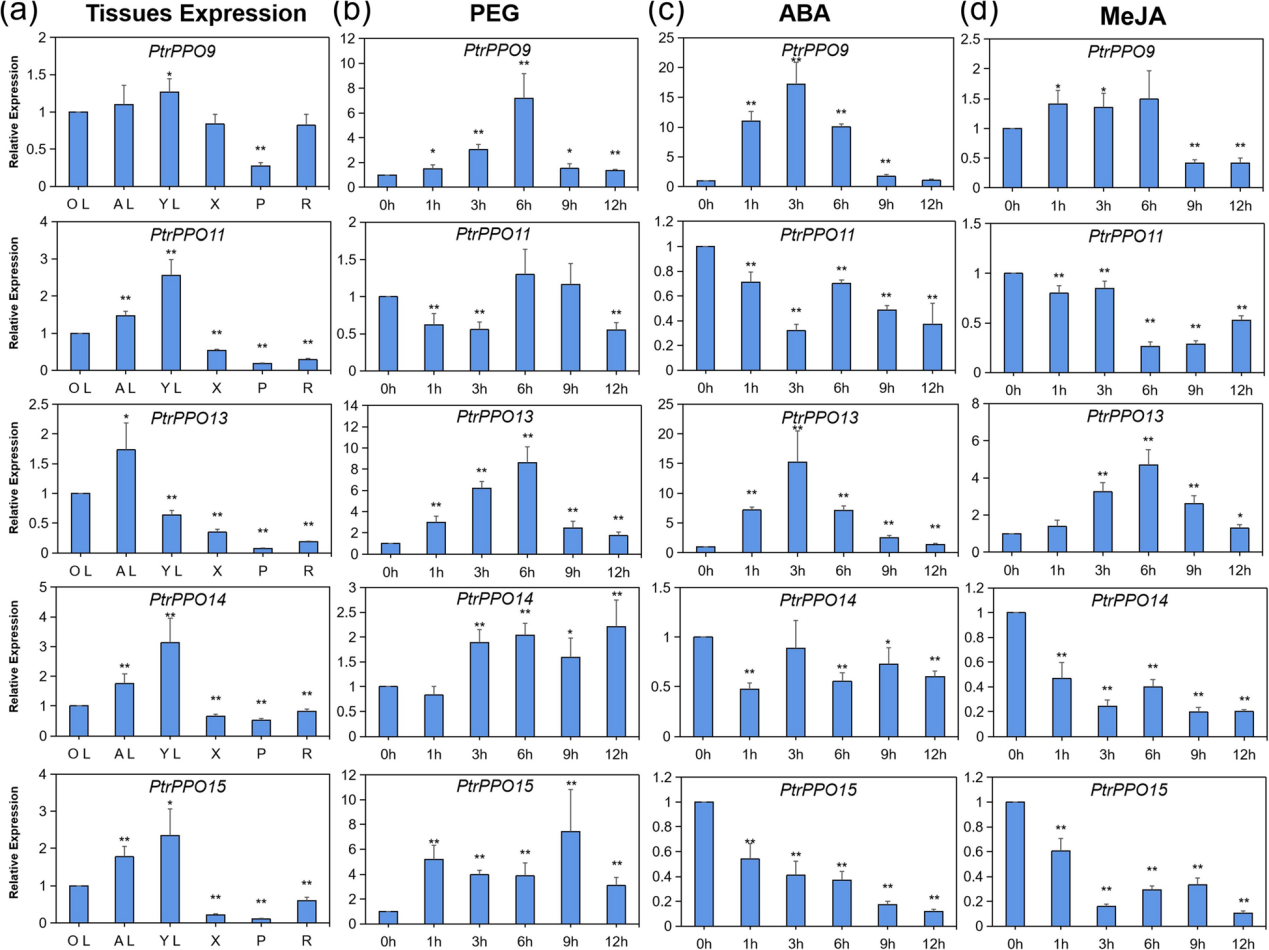
RT-qPCR analysis of the expression profile of *PPO* genes. **a** Transcript-level analysis of five *PtrPPOs* in various tissues. YL, young leaf; AL, adult leaf; OL, old leaf; P, phloem; X, xylem; R, root. Transcript levels of the five *PtrPPO*s in response to PEG (**b**), ABA (**c**), and MeJA (**d**) were determined using RT-qPCR. Values are means + SE (*n* = 20). Asterisks denote significant differences: *P ≤ 0.05; **P ≤ 0.01

The level of *PPO* gene expression in plants is closely related to stress and primarily reflects disease, insect and mechanical damage, and microorganism invasion [11, 22]. Results showed that the expression of five genes, i.e., *PtrPPO9/11/13/14/15,* was significantly induced or inhibited under conditions of stress. To verify the reliability of *PPO* gene expression in the transcriptome, the expression of 5 *PPO* (*PtrPPO9/11/13/14/15*) genes was investigated using RT-qPCR (Fig. 7a). Consistent with the previous results, most genes were expressed in young tissues. For example, except for *PtrPPO*13, which was preferentially expressed in mature leaves, the other four genes (*PtrPPO9/11/14/15*) were expressed primarily in the young leaves. Notably, *PtrPPO9/11/13/14/15* were expressed at high levels in leaves (compare to other tissues, such as xylem, phloem, and root).

To further validate whether *PPO* abundance was affected by abiotic stress and hormone treatment, the expression of 5 *PPO* members—from among the 18 *PtrPPOs* genes—was carefully investigated in response to PEG, ABA, and MeJA treatment using qRT-PCR (Fig. 7b–d). When poplar plants were treated with PEG, the expression of the other four genes was significantly induced except *PtrPPO11* (Fig. 7b)*.* PEG treatment significantly upregulated the expression of *PtrPPO9/13* that peaked 6 h after treatment. *PtrPPO14* was significantly upregulated after 3 h of treatment, and its expression level remained at approximately 2-fold. Furthermore, *PtrPPO15* was upregulated following PEG treatment, with a maximum expression of approximately 8-fold (compared to pre-treatment levels) after 9 h.

In response to ABA treatment, *PtrPPO9/13* showed similar expression patterns, with the expression of both genes significantly induced in response to ABA treatment, peaking after 3 h of treatment (approximately 20- and 15-fold pre-treatment levels, respectively) (Fig. 7c). However, the expression of *PtrPPO11/14/15* was significantly inhibited by ABA. In response to MeJA treatment, the expression of *PtrPPO13* was significantly induced, reaching a maximum value approximately 5-fold that of pre-treatment expression levels at 6 h of treatment, decreasing to approximately 3-fold at 9 h of treatment (Fig. 7d). In contrast, the expression of *PtrPPO11/14/15* was significantly inhibited by MeJA, whereas that of *PtrPPO9* was induced at 1 and 3 h but significantly inhibited at 9 and 12 h. Overall, each treatment may differentially induce and/or inhibit the expression of *PPO* genes in *Populus*.

### Identification and expression pattern analysis of potential upstream transcription factors (TFs) of *PtrPPOs*

**Fig. 8:**
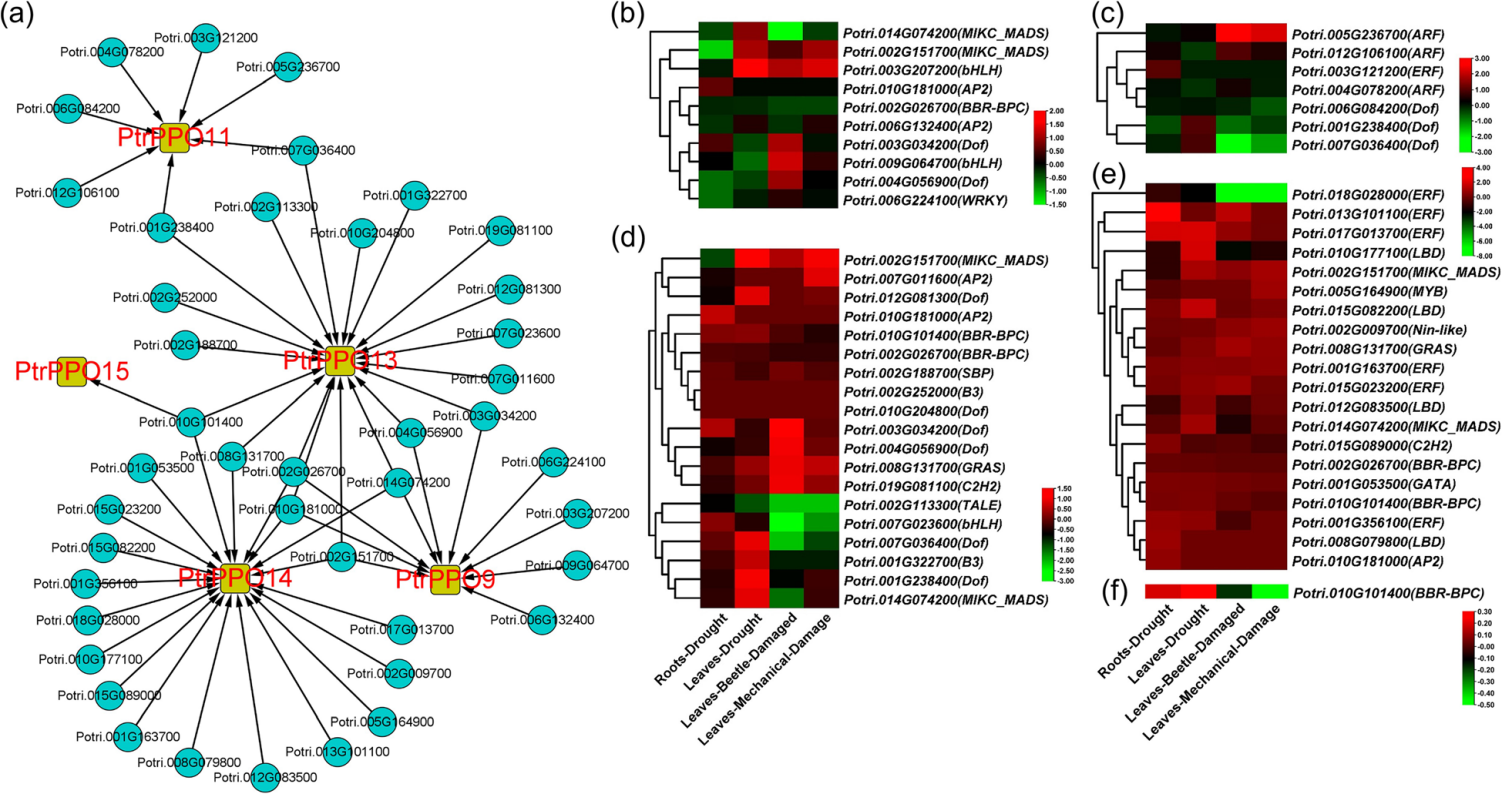
Bioinformatic analysis of potential upstream transcription factors (TFs) of *PtrPPOs*. **a** Molecular regulatory network analysis of TFs-*PtrPPO*9/11/13/14/15. The blue circles represent TFs upstream of *PtrPPO*s. The yellow square represents *PtrPPO*9/11/13/14/15. Cytoscape was used for visualization. Heat maps depicting the expression levels of potential upstream TFs of *PtrPPO9* (**b**)*, PtrPPO11* (**c**)*, PtrPPO13* (**d**)*, PtrPPO14* (**e**) and *PtrPPO15* (**f**) following drought-, beetle-, and mechanical damage. The color bar represents the range of maximum and minimum values of relative expressions in the heat map

To further understand how *PtrPPO9/11/13/14/15* function under stressful conditions, we identified potential upstream TFs of *PtrPPOs* using bioinformatics (Table S10). The regulatory network generated using Cytoscape (Fig. 8a) revealed that the expression of *PtrPPO*9/11/13/14/15 might be regulated by 10, 7, 19, 20, and 1 TFs, respectively. These TFs include Dof, ERF, BBR-BPC, MIKC_MADS, AP2, LBD, ARF, bHLH, B3, C2H2, GRAS, GATA, MYB, Nin-like, SBP, TALE, and WRKY family proteins. Moreover, *Potri.002G026700*, *Potri.002G151700*, *Potri.010G181000*, and *Potri.014G074200* may simultaneously regulate the expression of *PtrPPO*9/13/14 genes. We also found that *Potri.010G101400* may regulate the expression of three genes *PtrPPO*13/14/15.

We simultaneously evaluated the expression pattern of potential upstream TFs of *PtrPPO9/11/13/14/15* under stress conditions (Fig. 8b–f and Table S11). The potential upstream MIKC_MADS TFs (*Potri.002G151700*) and bHLH TFs (*Potri.003G207200*) of *PtrPPO9* were significantly upregulated in response to drought, beetle-, and mechanical-damage in poplar leaves (Fig. 8b). In addition, the expression of *Potri.003G034200* (Dof family protein), *Potri.009G064700* (bHLH family protein), and *Potri.004G056900* (Dof family protein) was significantly induced in beetle-damaged leaves (Fig. 8b). When poplar trees were exposed to beetle- and mechanical-damage, the expression of potential upstream ARF TFs (*Potri.005G236700* and *Potri.012G106100*) of *PtrPPO11* was significantly induced, and that of the potential upstream Dof TFs (*Potri.001G238400* and *Potri.007G036400*) of *PtrPPO11* was significantly inhibited (Fig. 8c).

Exposure of poplar trees to beetle- and mechanical-damage-induced stress resulted in the significant downregulation of the potential upstream TALE (*Potri.002G113300*), bHLH (*Potri.007G023600*), Dof (*Potri.007G036400*), and B3 TFs (*Potri.001G322700*) of *PtrPPO13* (Fig. 8d). Moreover, the expression of the potential upstream TFs of *PtrPPO14* was significantly induced by stress, except for ERF TFs (*Potri.018G028000*) (Fig. 8e). The expression of potential upstream BBR-BPC TF (*Potri.010G101400*) of *PtrPPO15* was significantly upregulated by drought stress in poplar leaves; however, it was significantly inhibited by beetle- and mechanical-damage induced stress (Fig. 8f).

## Discussion

As widespread copper metalloenzymes in plants, PPOs play an important role in plant growth, development, and stress tolerance [10, 20, 24]. With the development of whole genome sequencing, an increasing number of *PPO* genes have been identified in different plants, including apple, litchi, opium, potato, legumes, tea, mulberry, and tobacco [11]. However, the PPO family in the woody poplar plants is yet to be fully understood.

In the present study, 18 *PPO* genes were identified in poplar plants, and the characteristics of all products after replication, transcription, and translation were analyzed; additionally, their systematic evolution and expression models were constructed. In line with previous gene family studies [32], *PPO* genes in poplars were designated *PtrPPO1* to *PtrPPO18* according to their chromosomal locations (Table 1 and Figure S1). Except for PtrPPO10/12, all PtrPPO proteins had three conserved domains (KFDV, tyrosinase, and DWL), which may endow them with similar biological functions. It has been reported that the loss of protein structure could lead to different gene functions [33]. As both *PtrPPO10* and *PtrPPO12* only had one KFDV domain (Fig. 2d), they were clustered in the same branch (Fig. 1a and 2a), and the length of gDNA and the number of motifs in the protein were significantly lower than those in other genes (Fig. 2c). Furthermore, *PtrPPO10* and *PtrPPO12* only contained the PPO1_KFDV domain (Fig. 2d), suggesting the two proteins may have similar functions. As shown above, *PtrPPO10* and *PtrPPO12* cluster together when tissue expression patterns are investigated (Fig. 6a). The two genes have similar tissue expression patterns, and both are expressed at high levels in flowers and seeds, indicating that they may play a role in the formation of poplar flowers and seeds.

Most *PtrPPOs* have no introns, whereas some have three introns at most (Fig. 2b). Research has shown that introns play essential roles in the regulation of the transcriptome [34]. In order to respond quickly to stress, organisms need to stimulate the expression of genes, and gene structures with a few or no introns contribute to the rapid expression of mRNA [35]. For example, *PtrPPO7/8/1* genes lack introns, but contain UTRs; therefore, they can transcribe faster to form mRNA. In many plants, PPO genes respond quickly to both biological and abiotic stresses [11, 24].

We constructed an evolutionary tree from 25 model plants and divided it into 11 sections to further determine the origin and evolution of *PPO* genes. Consistent with the aforementioned results (Fig. 2a), PtrPPO protein was found in Groups V, VIII, IX, and XI (Fig. 1a). The phylogenetic tree analysis of family genes can clearly describe the evolutionary course of genes [36]. Because *Populus* (poplar) and *Salix* (willow) are both members of the family Salicaceae [37], most of their *PPO* genes are clustered in the same branch (IX). Notably, the *PPO* gene was not found in *Arabidopsis*, but four *PPO* genes were found in monocotyledonous *O. sativa* (Fig. 1b). It is worth noting that *PPO* genes are widely distributed in woody plants, especially in *P. trichocarpa* (18), *M. domestica* (15), and *S. purpurea* (10). No gene pairs have been found between *Arabidopsis* and poplar, whereas multiple gene pairs have been found between apple and poplar and between *S. purpurea* and poplar (Fig. 4), indicating that considerable differentiation and doubling of *PPOs* might have taken place during the evolution of perennial woody plants.

The increase in gene family members and the mechanism of genome evolution primarily depend on gene replication events, including tandem and segmented replications [38]. In the current study, 18 *PtrPPO* genes were unevenly distributed on 19 poplar chromosomes, and half of these *PtrPPO* genes were located on chromosome 1. This phenomenon of uneven distribution of chromosomes indicates that the change occurred before species differentiation. A total of three pairs of segmented replication genes and four tandem repeat genomes were detected in poplars, indicating that both tandem and segmental repeats contribute to the evolution of *PtrPPO* genes in *Populus* (Fig. 3). Previous gene family studies have shown that tandem repeat genes may have similar functions and expression patterns [34, 39]. For example, *PtrPPO12/13* has a similar expression level in all tissues, while *PtrPPO14/15* show a downward trend under adverse conditions (Fig. 6). Similar expression levels indicate similar functions and structures of tandem repeat genes.

The expression pattern of *PPO* genes in various tissues has been demonstrated in numerous species [22, 27, 28, 40]. Due to the difference in the number of PPOs in different species, there are no uniform gene expression profiles of *PPO* genes in plants. According to the RNA-seq data of poplar, some genes are preferentially expressed in young plant tissues and seeds (Fig. 6a), suggesting these tissues are more attractive to intruders. Consistent with the above results, fluorescence quantitative PCR results also showed that the *PPO* was expressed preferentially in young leaves than in other tissues (Fig. 7a). Research elsewhere has indicated that young leaves have higher PPO activity than old leaves [41].

The expression of *PPO* genes in plants is closely related to stress and mainly responds to insect and mechanical damage, diseases, and microorganism invasion [11, 22]. Transcriptome dataset analysis revealed that some *PPO* genes in poplar were significantly induced (*PtrPPO 9/13*) and inhibited (*PtrPPO 11/14/15*) by drought. A similar trend was observed using RT-qPCR. PEG and ABA treatments significantly upregulated *PtrPPO* 9/13 expression (Fig. 7b and c). By analyzing the promoters of *PtrPPO* 9/13, we found that there were multiple abscisic acid responsiveness elements in these two promoters (Fig. 5). *PtrPPO9* was significantly upregulated under conditions of PEG and ABA treatment than under MeJA treatment, indicating that *PtrPPO9* had a higher probability of being induced by abiotic stress. In addition, *PPO* genes in *Nicotiana tabacum* and *Glycine max* were significantly induced by ABA [24, 42]. Transcriptome analysis verified that *PtrPPO*13 was significantly upregulated, and *PtrPPO*11/14/15 was significantly inhibited in response to mechanical injury and damage caused by the poplar borer (beetle; Fig. 6b). MeJA is involved in signal transduction during insect attack and plant mechanical damage [23, 24]. MeJA responsiveness elements were identified on the *PtrPPO13* promoter*.* Additionally, RT-qPCR revealed that *PtrPPO13* was significantly induced by MeJA (Fig. 7d). *PtrPPO13* was significantly induced by PEG, ABA, and MeJA treatments, reaching a peak at 3 or 6 h, suggesting that *PtrPPO13* might be involved in biological and abiotic stress. Compared with the expression profiles revealed by RNA-seq data, the patterns of qRT-PCR expression were dissimilar. Various reasons might cause the observed differences in expressions.

In recent years, promoters, TFs, and microRNAs (miRNAs) have been found to regulate *PPO* response to stress in plants [11]. miR528, miR12112, and miR058 from Banana, *Salvia miltiorrhiza,* and Grapevine can silence *PPO* genes and participate in cold stress and MeJA response, respectively [23, 43–45]. Similarly, TFs play a crucial role in the transcriptional regulation of genes [46]. The potential upstream TFs of *PtrPPOs* were identified using bioinformatics (Fig. 8a). Compared with the previous results (Fig. 6b), some potential upstream TFs of *PtrPPOs* were also induced significantly in stress (Fig. 8a-f). Recently, it has been reported that MnMYB3R1 in Mulberry can combine with the MSA element on the *MnPPO1* promoter and then regulate *MnPPO1* to increase plant drought resistance [28]. In conclusion, PPOs play a critical role in the regulation of stress response.

The finding of the current study allows us to infer the functional roles of *PPO* genes in poplar plants. Comprehensive analysis is useful to select candidate *PPO* genes for further functional characterization, while genetic improvement of stress resistance in forest trees provides genetic resources.

## Conclusions

To summarize, whole-genome analysis of the poplar PPO family was accomplished, and 18 *PtrPPO* genes were identified. Bioinformatics and qRT-PCR were then used to analyze the gene structure, phylogeny, chromosomal localization, gene replication, *cis*-elements, and expression patterns of *PtrPPOs*. Finally, we found that *PPO* genes were preferentially expressed in young plant tissues and fruits, and some genes could be significantly induced by PEG, ABA, and MeJA, indicating that *PPO* plays an integral part in the stress resistance of poplars. This research will help to explore the function of *PPO* genes in *Populus*.

## Methods

### Genome-wide identification and retrieval of *PPO* genes in *P. trichocarpa*

The extraction and identification of poplar family members were conducted according to a previously described method [47]. The genome data of *A. thaliana* (Athaliana_447_TAIR10.fa.gz), *O. sativa* (Osativa_323_v7.0.fa.gz), and *P. trichocarpa* (Ptrichocarpa_533_v4.0.fa.gz) were obtained from the Phytozome database (https://phytozome.jgi.doe.gov/pz/portal.html) [48]. In addition, BLAST and HMMER were used to identify 18 PPO proteins with conserved structures in poplar plants. Redundant sequences were manually discarded. Furthermore, the conservative structure, molecular weights, isoelectric points, and hydrophilicity analysis of the identified PPOs were conducted using SMART on the ExPasy server (https://web.expasy.org/protparam/) [32].

### Evolutionary relationships of *PPO* genes

A total of 138 PPO proteins from *Aquilegia coerulea, Ananas comosus, Amaranthus hypochondriacus, Carica papaya, Cucumis sativus, Eucalyptus grandis, Gossypium raimondii, Kalanchoe fedtschenkoi, Kalanchoe laxiflora, Linum usitatissimum, Musa acuminata, Malus domestica, Mimulus guttatus, Marchantia polymorpha, Oryza sativa, Oropetium thomaeum, Populus trichocarpa, Panicum virgatum, Ricinus communis, Sorghum bicolor, Sphagnum fallax, Solanum lycopersicum, Spirodela polyrhiza, Salix purpurea,* and *Solanum tuberosum* were obtained from the Phytozome database [49]. They all had PPO-KFDV conserved domains. The amino acids of all PPO target sequences were analyzed using ClustalX, and then the phylogenetic tree was constructed using the Neighbor-Joining (NJ) method in MEGA7.0. The accession numbers and gene names are all shown in Table S2.

### Analysis of gene and protein structures and motifs

The Gene Structure Display Server (http://gsds.cbi.pku.edu.cn/) [50] was used to analyze the introns, exons, and UTRs of the 18 poplar *PPO* genes. The conserved motifs and domains of candidate PPO proteins were identified using MEME (https://meme-suite.org/meme/tools/meme) [51] and Conserved Domains Database (CDD) (https://www.ncbi.nlm.nih.gov/Structure/bwrpsb/bwrpsb.cgi?) [52], respectively. Finally, the conserved motifs and structures of all PPO proteins in poplar were drawn using TBtools (https://github.com/CJ-Chen/TBtools). Previously described specific parameter settings were used [53].

### Chromosomal locations and gene duplication

The chromosomal positions of *PtrPPOs* were collected from the Phytozome database [49]. MCScanX was used to analyze *PPO* gene duplication events [54]. TBtools was used to display the locations and collinearity of *PtrPPO* genes.

### Analysis of cis-regulatory elements

The promoter sequences (2000 bp upstream of the start codon) of *PPOs* were analyzed online using Plant-CARE (http://bioinformatics.psb.ugent.be/webtools/plantcare/html/) [55], and all *cis*-regulatory elements related to hormones and stress were identified.

### Transcriptomic data sets to analyze the expression patterns of *PPOs*

To evaluate *PtrPPOs* gene expression profiles, the publicly available transcriptomic data were obtained from PopGenIE (https://popgenie.org/) [56]. In the current study, transcriptome data were collected from stressed plants (drought, mechanical damage, insect beetle damage) and 15 different plant tissues (non-girdled twigs, dormant flowers, expanded flowers, mature leaves, expanding flowers, whole-sucker suckers, mature petiole, prechilling buds, dormant buds, freshly expanded leaves, non-girdled leaves, girdled leaves, mature seeds, young expanding leaves, and dormant cambium phloem) during the growth and development of *P. trichocarpa.* The relative expression of *PtrPPOs* was displayed as a heat map generated using TBtools [53].

### Plant materials and treatments

*Populus* (*Populus trichocarpa*) plant seeds were acquired from Sichuan Agricultural University trail *Populus* planting base [48]. One-year-old *Populus trichocarpa* seedlings were planted in a plastic plot (16.0 h light; 20–25 °C; 70% air humidity) in Wenjiang, Chengdu, China (30°70′ N, 103°85′ E, 537.11 m above sea level). The plants were watered with 1 L Hoagland nutrient solution every 2 weeks for 2 months before treatment [46]. Previously, PEG, ABA, and MeJA treatments have been used to investigate the gene responses to abiotic stresses in plants [48, 57, 58]. Sixty-day-old seedlings were treated with PEG, ABA, and MeJA. At least five biological replicates were used for each treatment. For PEG treatments, similarly grown *P. trichocarpa* seedlings (40–50-cm height, with 30–35 leaves) were treated with 15% PEG6000 for 0, 1, 3, 6, 9, and 12 h. For phytohormone analysis, similarly grown *P. trichocarpa* seedlings were treated with a solution containing 200 μM ABA (Sigma-Aldrich, Santa Clara, CA, USA) and MeJA (Sigma-Aldrich), for 0, 1, 3, 6, 9, and 12 h. The leaves were separated from the plant at the different processing time points, rapidly frozen in liquid nitrogen, and stored in an ultra-low temperature freezer (Thermo Fisher Scientific, Waltham, MA, USA).

Poplar tissues and organs were sampled in accordance with a previously described method [48]. Plant tissues and organs were sampled under stress-free conditions; each plant tissue and organ had at least five biological replicates. We simultaneously collected different *P. trichocarpa* organs and tissues, including young, mature, and old leaves, xylem, phloem, and roots, and immediately immersed them in liquid nitrogen.

### RNA extraction and quantitative real-time (qRT-PCR) analysis

Approximately 1 g of tissue was isolated for total RNA extraction. Total RNA was prepared using the plant total RNA extraction kit (Aidlab, Beijing, China) according to the manufacturer’s protocol, and 2 μg of total RNA was reverse transcribed into cDNA using the Tiangen Fast Quant RT Kit (Tiangen Biotech Co. Ltd., Beijing, China). qRT-PCR was conducted as per a previously described protocol [59]. Based on the target gene fragment, Primer Premier 6.0 was used to design primers online; all primers used are listed in Table S1. At least 20 replicates (5 biological replicates × 4 technical replicates) were used per experiment.

### Bioinformatic analysis of potential TFs upstream of *PtrPPOs*

The plant regulatory network database PlantRegMap (http://plantregmap.gao-lab.org/) [60] was used to identify potential upstream transcriptional factors of *PtrPPO9/11/13/14/15*. The molecular network regulation map of target genes and upstream transcription factors was drawn using Cytoscape [61]. Transcriptome data for upstream transcription factors under stress conditions (drought and mechanical and insect damage) were obtained from PopGenIE (https://popgenie.org/) [56] and the heat maps were drawn using TBtools.

### Availability of data and materials

The datasets generated and/or analyzed during the current study are available in this article and additional files. The nucleotide and protein sequences of PPO-related genes in *Aquilegia coerulea, Ananas comosus, Amaranthus hypochondriacus, Carica papaya, Cucumis sativus, Eucalyptus grandis, Gossypium raimondii, Kalanchoe fedtschenkoi, Kalanchoe laxiflora, Linum usitatissimum, Musa acuminata, Malus domestica, Mimulus guttatus, Marchantia polymorpha, Oryza sativa, Oropetium thomaeum, Populus trichocarpa, Panicum virgatum, Ricinus communis, Sorghum bicolor, Sphagnum fallax, Solanum lycopersicum, Spirodela polyrhiza, Salix purpurea,* and *Solanum tuberosum* are available in the Phytozome v12.1 database (JGI, https://phytozome.jgi.doe.gov/pz/portal.html).

### Statistical analyses

Microsoft Excel 2020 (Microsoft Corporation, Redmond, WA, USA) and SPSS v.17.0 (SPSS Inc., Chicago, IL, USA) were used to analyze the experimental data. Both one-way analysis of variance was used to determine the significance of the differences among treatments. Student’s *t*-test was used to calculate *P*-values (*P < 0.05; **P < 0.01). The data were normalized, and all samples were normally distributed in terms of homogeneity of variance.

## Supplementary Information

**Additional file 1.**

## Data Availability

All data supporting the conclusions of this article are provided within the article and its additional files. The genomics sequences data of *A. thaliana*, *O. sativa*, and *P. trichocarpa* are available in the Phytozome database (https://phytozome.jgi.doe.gov/pz/portal.html). The public RNA-seq data are available on PopGenIE (https://popgenie.org/gene?id=Potri.001G388900).

## Abbreviations

CDS: Coding sequence
AA: Amino acid
MW: Molecular mass (kDa)
GRAVY: Grand average of hydropathicity
PI: Theoretical isoelectric point
