# Supplementary material for: Genome-wide investigation and expression profiling of polyphenol oxidase (PPO) family genes uncover likely functions in organ development and stress responses in Populus trichocarpa
Source: BMC Genomics. 2021 Oct 8;22:731. doi: 10.1186/s12864-021-08028-9 (PMC8501708; doi:10.1186/s12864-021-08028-9)
Supplement: Supplementary file 1 — Additional file 1. [file 12864_2021_8028_MOESM1_ESM.docx]

**Table S1. Primer sequences used RT-PCR**

| **Gene Name** | **Forward/Reverse primers** | **Tm/℃** | **Product length** |
| --- | --- | --- | --- |
| *PtrPPO9* | F:TCCCTCATCTTATAAAAAGGGCCTC | 60.16 | 256 |
|  | R:AGGACATCCCTTCTCGTGGA | 59.96 |  |
| *PtrPPO11* | F:GCAGTGAAGGTGTGAGTGGC | 60.03 | 298 |
|  | R:TACGGGCGACTTCTAGTGGA | 60.04 |  |
| *PtrPPO13* | F:CGAACTGACCTGACTGACCC | 60.04 | 159 |
|  | R:TGGCCTGGATTGTAGCCAAG | 60.03 |  |
| *PtrPPO14* | F:CCTGTTGTCATCTCTGGCGTTA | 60.35 | 91 |
|  | R:ACATGTTGTTGCCCAATAATAGCA | 59.53 |  |
| *PtrPPO15* | F:GCTTTGGTGCCCAGGTCTAA | 60.25 | 88 |
|  | R:AGGTGGACATTCACTCTTTAACAA | 58.31 |  |

**Table S2. Accession numbers of gene**

| **Species** | **Name** | **Accession numbers** |
| --- | --- | --- |
| *Aquilegia coerulea* | *AqcPPO1* | Aqcoe1G318200 |
| *Aquilegia coerulea* | *AqcPPO2* | Aqcoe1G318300 |
| *Aquilegia coerulea* | *AqcPPO3* | Aqcoe1G318400 |
| *Aquilegia coerulea* | *AqcPPO4* | Aqcoe2G240400 |
| *Ananas comosus* | *AcPPO1* | Aco014845 |
| *Ananas comosus* | *AcPPO2* | Aco014847 |
| *Ananas comosus* | *AcPPO3* | Aco014848 |
| *Ananas comosus* | *AcPPO4* | Aco014850 |
| *Amaranthus hypochondriacus* | *AhPPO1* | AHYPO_004163 |
| *Amaranthus hypochondriacus* | *AhPPO2* | AHYPO_004165 |
| *Amaranthus hypochondriacus* | *AhPPO3* | AHYPO_004166 |
| *Amaranthus hypochondriacus* | *AhPPO4* | AHYPO_008370 |
| *Amaranthus hypochondriacus* | *AhPPO5* | AHYPO_011354 |
| *Amaranthus hypochondriacus* | *AhPPO6* | AHYPO_019806 |
| *Carica papaya* | *CpPPO1* | evm.TU.supercontig_27.43 |
| *Carica papaya* | *CpPPO2* | evm.TU.supercontig_62.169 |
| *Cucumis sativus* | *CsPPO1* | Cucsa.126490 |
| *Eucalyptus grandis* | *EgPPO1* | Eucgr.D00657 |
| *Eucalyptus grandis* | *EgPPO2* | Eucgr.D00659 |
| *Eucalyptus grandis* | *EgPPO3* | Eucgr.L00689 |
| *Gossypium raimondii* | *GrPPO1* | Gorai.006G086400 |
| *Gossypium raimondii* | *GrPPO2* | Gorai.010G151800 |
| *Gossypium raimondii* | *GrPPO3* | Gorai.010G152000 |
| *Gossypium raimondii* | *GrPPO4* | Gorai.010G254900 |
| *Kalanchoe fedtschenkoi* | *KfPPO1* | Kaladp0010s0069 |
| *Kalanchoe fedtschenkoi* | *KfPPO2* | Kaladp1262s0012 |
| *Kalanchoe laxiflora* | *KlPPO1* | Kalax.0092s0048 |
| *Kalanchoe laxiflora* | *KlPPO2* | Kalax.0239s0002 |
| *Kalanchoe laxiflora* | *KlPPO3* | Kalax.0658s0017 |
| *Kalanchoe laxiflora* | *KlPPO4* | Kalax.0856s0002 |
| *Linum usitatissimum* | *LuPPO1* | Lus10038309.g |
| *Musa acuminata* | *MaPPO1* | GSMUA_Achr7G03560_001 |
| *Musa acuminata* | *MaPPO2* | GSMUA_Achr8G34370_001 |
| *Musa acuminata* | *MaPPO3* | GSMUA_AchrUn_randomG22730_001 |
| *Musa acuminata* | *MaPPO4* | GSMUA_AchrUn_randomG22740_001 |
| *Musa acuminata* | *MaPPO5* | GSMUA_AchrUn_randomG25220_001 |
| *Malus domestica* | *MdPPO1* | MDP0000007676 |
| *Malus domestica* | *MdPPO2* | MDP0000007682 |
| *Malus domestica* | *MdPPO3* | MDP0000173059 |
| *Malus domestica* | *MdPPO4* | MDP0000207799 |
| *Malus domestica* | *MdPPO5* | MDP0000221498 |
| *Malus domestica* | *MdPPO6* | MDP0000234782 |
| *Malus domestica* | *MdPPO7* | MDP0000298729 |
| *Malus domestica* | *MdPPO8* | MDP0000317364 |
| *Malus domestica* | *MdPPO9* | MDP0000478750 |
| *Malus domestica* | *MdPPO10* | MDP0000500159 |
| *Malus domestica* | *MdPPO11* | MDP0000511406 |
| *Malus domestica* | *MdPPO12* | MDP0000609966 |
| *Malus domestica* | *MdPPO13* | MDP0000699845 |
| *Malus domestica* | *MdPPO14* | MDP0000709073 |
| *Malus domestica* | *MdPPO15* | MDP0000744636 |
| *Mimulus guttatus* | *MgPPO1* | Migut.M00642 |
| *Mimulus guttatus* | *MgPPO2* | Migut.M01175 |
| *Marchantia polymorpha* | *MpPPO1* | Mapoly0024s0005 |
| *Marchantia polymorpha* | *MpPPO2* | Mapoly0038s0001 |
| *Marchantia polymorpha* | *MpPPO3* | Mapoly0038s0002 |
| *Marchantia polymorpha* | *MpPPO4* | Mapoly0038s0003 |
| *Marchantia polymorpha* | *MpPPO5* | Mapoly0038s0004 |
| *Marchantia polymorpha* | *MpPPO6* | Mapoly0038s0112 |
| *Marchantia polymorpha* | *MpPPO7* | Mapoly0091s0043 |
| *Marchantia polymorpha* | *MpPPO8* | Mapoly0121s0018 |
| *Marchantia polymorpha* | *MpPPO9* | Mapoly0121s0019 |
| *Marchantia polymorpha* | *MpPPO10* | Mapoly0145s0006 |
| *Marchantia polymorpha* | *MpPPO11* | Mapoly0145s0008 |
| *Marchantia polymorpha* | *MpPPO12* | Mapoly0145s0009 |
| *Marchantia polymorpha* | *MpPPO13* | Mapoly0145s0011 |
| *Marchantia polymorpha* | *MpPPO14* | Mapoly0145s0012 |
| *Marchantia polymorpha* | *MpPPO15* | Mapoly0237s0004 |
| *Marchantia polymorpha* | *MpPPO16* | Mapoly0237s0005 |
| *Marchantia polymorpha* | *MpPPO17* | Mapoly0391s0001 |
| *Oryza sativa* | *OsPPO1* | LOC_Os01g58100 |
| *Oryza sativa* | *OsPPO2* | LOC_Os04g53250 |
| *Oryza sativa* | *OsPPO3* | LOC_Os04g53290 |
| *Oryza sativa* | *OsPPO4* | LOC_Os04g53300 |
| *Oropetium thomaeum* | *OtPPO1* | Oropetium_20150105_01740 |
| *Oropetium thomaeum* | *OtPPO2* | Oropetium_20150105_14050 |
| *Oropetium thomaeum* | *OtPPO3* | Oropetium_20150105_18562 |
| *Populus trichocarpa* | *PtrPPO1* | Potri.001G387900 |
| *Populus trichocarpa* | *PtrPPO2* | Potri.001G388000 |
| *Populus trichocarpa* | *PtrPPO3* | Potri.001G388100 |
| *Populus trichocarpa* | *PtrPPO4* | Potri.001G388200 |
| *Populus trichocarpa* | *PtrPPO5* | Potri.001G388300 |
| *Populus trichocarpa* | *PtrPPO6* | Potri.001G388400 |
| *Populus trichocarpa* | *PtrPPO7* | Potri.001G388600 |
| *Populus trichocarpa* | *PtrPPO8* | Potri.001G388800 |
| *Populus trichocarpa* | *PtrPPO9* | Potri.001G388900 |
| *Populus trichocarpa* | *PtrPPO10* | Potri.004G038500 |
| *Populus trichocarpa* | *PtrPPO11* | Potri.004G156500 |
| *Populus trichocarpa* | *PtrPPO12* | Potri.011G047200 |
| *Populus trichocarpa* | *PtrPPO13* | Potri.011G047300 |
| *Populus trichocarpa* | *PtrPPO14* | Potri.011G108200 |
| *Populus trichocarpa* | *PtrPPO15* | Potri.011G108300 |
| *Populus trichocarpa* | *PtrPPO16* | Potri.T061900 |
| *Populus trichocarpa* | *PtrPPO17* | Potri.T062100 |
| *Populus trichocarpa* | *PtrPPO18* | Potri.T062200 |
| *Panicum virgatum* | *PvPPO1* | Pavir.J28528 |
| *Panicum virgatum* | *PvPPO2* | Pavir.Fb00567 |
| *Ricinus communis* | *RcPPO1* | 29851.t000009 |
| *Sorghum bicolor* | *SbPPO1* | Sobic.003G310400 |
| *Sorghum bicolor* | *SbPPO2* | Sobic.006G181300 |
| *Sorghum bicolor* | *SbPPO3* | Sobic.007G068300 |
| *Sorghum bicolor* | *SbPPO4* | Sobic.007G068500 |
| *Sorghum bicolor* | *SbPPO5* | Sobic.007G068700 |
| *Sorghum bicolor* | *SbPPO6* | Sobic.010G192700 |
| *Sphagnum fallax* | *SfPPO1* | Sphfalx0022s0057 |
| *Sphagnum fallax* | *SfPPO2* | Sphfalx0048s0110 |
| *Sphagnum fallax* | *SfPPO3* | Sphfalx0310s0011 |
| *Sphagnum fallax* | *SfPPO4* | Sphfalx0213s0034 |
| *Solanum lycopersicum* | *SlPPO1* | Solyc02g078650.2 |
| *Solanum lycopersicum* | *SlPPO2* | Solyc08g074620.1 |
| *Solanum lycopersicum* | *SlPPO3* | Solyc08g074630.1 |
| *Solanum lycopersicum* | *SlPPO4* | Solyc08g074640.1 |
| *Solanum lycopersicum* | *SlPPO5* | Solyc08g074650.2 |
| *Solanum lycopersicum* | *SlPPO6* | Solyc08g074680.2 |
| *Solanum lycopersicum* | *SlPPO7* | Solyc08g074690.2 |
| *Spirodela polyrhiza* | *SpiPPO1* | Spipo0G0115000 |
| *Spirodela polyrhiza* | *SpiPPO2* | Spipo24G0018500 |
| *Spirodela polyrhiza* | *SpiPPO3* | Spipo24G0018800 |
| *Spirodela polyrhiza* | *SpiPPO4* | Spipo24G0019000 |
| *Spirodela polyrhiza* | *SpiPPO5* | Spipo25G0004100 |
| *Spirodela polyrhiza* | *SpiPPO6* | Spipo25G0004200 |
| *Salix* *purpurea* | *SpPPO1* | SapurV1A.0044s0470 |
| *Salix purpurea* | *SpPPO2* | SapurV1A.0064s0010 |
| *Salix purpurea* | *SpPPO3* | SapurV1A.0064s0020 |
| *Salix purpurea* | *SpPPO4* | SapurV1A.0064s0030 |
| *Salix purpurea* | *SpPPO5* | SapurV1A.0064s0040 |
| *Salix purpurea* | *SpPPO6* | SapurV1A.0064s0050 |
| *Salix purpurea* | *SpPPO7* | SapurV1A.0064s0060 |
| *Salix purpurea* | *SpPPO8* | SapurV1A.0299s0070 |
| *Salix purpurea* | *SpPPO9* | SapurV1A.0721s0060 |
| *Salix purpurea* | *SpPPO10* | SapurV1A.0737s0010 |
| *Solanum tuberosum* | *StPPO1* | PGSC0003DMG400018913 |
| *Solanum tuberosum* | *StPPO2* | PGSC0003DMG400018914 |
| *Solanum tuberosum* | *StPPO3* | PGSC0003DMG400018916 |
| *Solanum tuberosum* | *StPPO4* | PGSC0003DMG400018917 |
| *Solanum tuberosum* | *StPPO5* | PGSC0003DMG400018924 |
| *Solanum tuberosum* | *StPPO6* | PGSC0003DMG400018925 |
| *Solanum tuberosum* | *StPPO7* | PGSC0003DMG400029576 |

**Table S3.** **Amino acid conserved domain**

| **Gene ID** | **Pfam ID** | **From** | **To** | **Short name** |
| --- | --- | --- | --- | --- |
| Q#1 - >PtPPO1 | pfam12143 | 453 | 578 | PPO1_KFDV |
| Q#1 - >PtPPO1 | pfam00264 | 159 | 367 | Tyrosinase |
| Q#1 - >PtPPO1 | pfam12142 | 377 | 428 | PPO1_DWL |
| Q#2 - >PtPPO2 | pfam12142 | 47 | 97 | PPO1_DWL |
| Q#2 - >PtPPO2 | pfam12143 | 110 | 189 | PPO1_KFDV |
| Q#2 - >PtPPO2 | pfam00264 | 5 | 44 | Tyrosinase |
| Q#3 - >PtPPO3 | pfam12143 | 393 | 518 | PPO1_KFDV |
| Q#3 - >PtPPO3 | pfam00264 | 91 | 307 | Tyrosinase |
| Q#3 - >PtPPO3 | pfam12142 | 317 | 368 | PPO1_DWL |
| Q#4 - >PtPPO4 | pfam12143 | 453 | 578 | PPO1_KFDV |
| Q#4 - >PtPPO4 | pfam00264 | 159 | 367 | Tyrosinase |
| Q#4 - >PtPPO4 | pfam12142 | 377 | 428 | PPO1_DWL |
| Q#5 - >PtPPO5 | pfam12143 | 313 | 438 | PPO1_KFDV |
| Q#5 - >PtPPO5 | pfam12142 | 237 | 287 | PPO1_DWL |
| Q#5 - >PtPPO5 | pfam00264 | 74 | 227 | Tyrosinase |
| Q#6 - >PtPPO6 | pfam12143 | 453 | 578 | PPO1_KFDV |
| Q#6 - >PtPPO6 | pfam00264 | 159 | 367 | Tyrosinase |
| Q#6 - >PtPPO6 | pfam12142 | 377 | 428 | PPO1_DWL |
| Q#7 - >PtPPO7 | pfam12143 | 453 | 578 | PPO1_KFDV |
| Q#7 - >PtPPO7 | pfam00264 | 159 | 367 | Tyrosinase |
| Q#7 - >PtPPO7 | pfam12142 | 377 | 428 | PPO1_DWL |
| Q#8 - >PtPPO8 | pfam12143 | 453 | 578 | PPO1_KFDV |
| Q#8 - >PtPPO8 | pfam00264 | 159 | 367 | Tyrosinase |
| Q#8 - >PtPPO8 | pfam12142 | 377 | 428 | PPO1_DWL |
| Q#9 - >PtPPO9 | pfam12143 | 482 | 603 | PPO1_KFDV |
| Q#9 - >PtPPO9 | pfam00264 | 193 | 400 | Tyrosinase |
| Q#9 - >PtPPO9 | pfam12142 | 407 | 455 | PPO1_DWL |
| Q#10 - >PtPPO10 | pfam12143 | 118 | 244 | PPO1_KFDV |
| Q#11 - >PtPPO11 | pfam00264 | 140 | 357 | Tyrosinase |
| Q#11 - >PtPPO11 | pfam12143 | 453 | 585 | PPO1_KFDV |
| Q#11 - >PtPPO11 | pfam12142 | 363 | 413 | PPO1_DWL |
| Q#12 - >PtPPO12 | pfam12143 | 115 | 240 | PPO1_KFDV |
| Q#13 - >PtPPO13 | pfam12143 | 460 | 587 | PPO1_KFDV |
| Q#13 - >PtPPO13 | pfam00264 | 160 | 368 | Tyrosinase |
| Q#13 - >PtPPO13 | pfam12142 | 374 | 425 | PPO1_DWL |
| Q#14 - >PtPPO14 | pfam12143 | 430 | 522 | PPO1_KFDV |
| Q#14 - >PtPPO14 | pfam00264 | 184 | 348 | Tyrosinase |
| Q#14 - >PtPPO14 | pfam12142 | 358 | 408 | PPO1_DWL |
| Q#15 - >PtPPO15 | pfam12143 | 433 | 560 | PPO1_KFDV |
| Q#15 - >PtPPO15 | pfam00264 | 145 | 351 | Tyrosinase |
| Q#15 - >PtPPO15 | pfam12142 | 361 | 411 | PPO1_DWL |
| Q#16 - >PtPPO16 | pfam12143 | 453 | 578 | PPO1_KFDV |
| Q#16 - >PtPPO16 | pfam00264 | 159 | 367 | Tyrosinase |
| Q#16 - >PtPPO16 | pfam12142 | 377 | 428 | PPO1_DWL |
| Q#17 - >PtPPO17 | pfam12143 | 417 | 542 | PPO1_KFDV |
| Q#17 - >PtPPO17 | pfam00264 | 159 | 331 | Tyrosinase |
| Q#17 - >PtPPO17 | pfam12142 | 341 | 392 | PPO1_DWL |
| Q#18 - >PtPPO18 | pfam12143 | 453 | 578 | PPO1_KFDV |
| Q#18 - >PtPPO18 | pfam00264 | 159 | 367 | Tyrosinase |
| Q#18 - >PtPPO18 | pfam12142 | 377 | 428 | PPO1_DWL |

**Table S4. Amino acid sequence encode and SeqLogo of the Motif**

| **Motif ID** | **Amino acid sequence encode** | **SeqLogo** |
| --- | --- | --- |
| Motif_1 | ENMGNFYSAGRDPIFYCHHSNVDRMW | 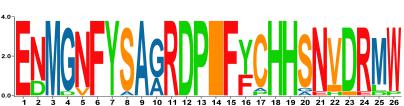 |
| Motif_2 | LDKVISVEVSRPKKSRSATEKEDEDEVLVIEGIEYEENQLIKFDVLVNDE | 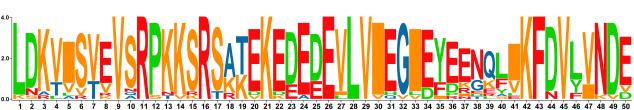 |
| Motif_3 | QIHFSWLFFPWHRLYLYYFERILGKLIDDPTFALPFWNWDAPAGMQMPAI | 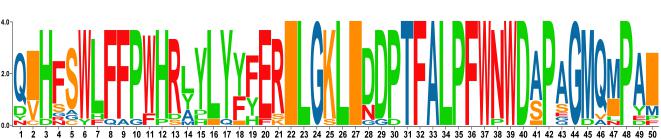 |
| Motif_4 | DWLNSEFLFWDENKELVRVKVKDTLDTKKLRYGFQDVPIPWLKTRATPKL | 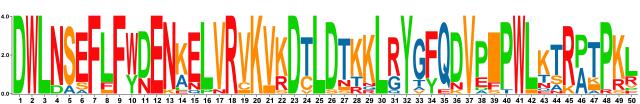 |
| Motif_5 | MKSLPDDDPRSFKSQANVHCAYCDGAYHQ | 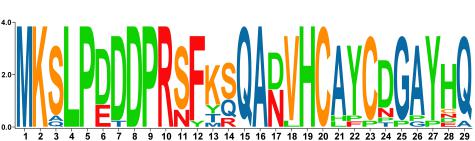 |
| Motif_6 | DPSPGMGTIETTPHTQIHYWTGDPNQTNG | 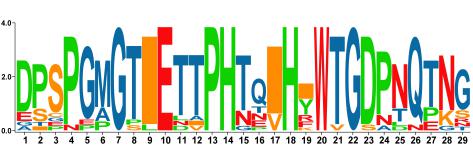 |
| Motif_7 | YANPIAPPDITQCELVTLPTESDPSNCCPPTSTKIKNFEFPSASSPMRIR | 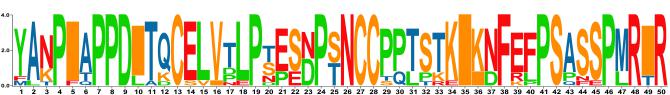 |
| Motif_8 | ANPDPAKAEELYASNLNVMYRQMVSGATKPTLFFGKPYRAG | 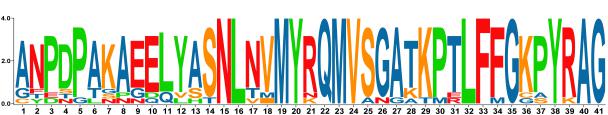 |
| Motif_9 | AKKSKTTMVLGITGLLEDLEAEGDDTLVV | 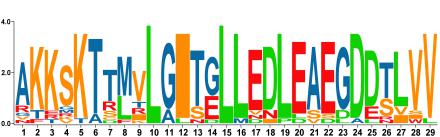 |
| Motif_10 | KTHRVSRIKKPNRHNIPIVSCKSGKNDHEQNPATRRDLLIG | 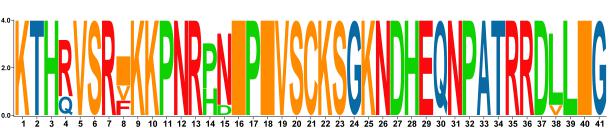 |

**Table S5. Collinear genes among different species**

| **Ptr-Sp** | **Ptr-Md** | **Ptr-Os** | **Ptr-At** |
| --- | --- | --- | --- |
| *PtPPO1(Potri.001G387900)* | *PtPPO10(Potri.004G038500)* |  |  |
| *PtPPO10(Potri.004G038500)* | *PtPPO12(Potri.011G047200)* |  |  |
| *PtPPO11(Potri.004G156500)* | *PtPPO13(Potri.011G047300)* |  |  |
| *PtPPO12(Potri.011G047200)* |  |  |  |
| *PtPPO14(Potri.011G108200)* |  |  |  |

**Table S6. Information of functional elements in PPOs gene**

| **GeneID** | **Element** | **Sequence** | **Description** |
| --- | --- | --- | --- |
| *PtrPPO1* | P-box | CCTTTTG | gibberellin-responsive element |
|  | TC-rich repeats | ATTCTCTAAC | defense and stress responsiveness |
|  | O2-site | GATGATGTGG | zein metabolism regulation |
|  | CGTCA-motif | CGTCA | MeJA-responsiveness |
|  | TCA-element | CCATCTTTTT | salicylic acid responsiveness |
|  | ABRE | ACGTG | abscisic acid responsiveness |
|  | TGACG-motif | TGACG | MeJA-responsiveness |
| *PtrPPO2* | ABRE | ACGTG | abscisic acid responsiveness |
|  | TGACG-motif | TGACG | MeJA-responsiveness |
|  | CGTCA-motif | CGTCA | MeJA-responsiveness |
|  | MBS | CAACTG | drought-inducibility |
|  | MBS | CAACTG | drought-inducibility |
|  | TC-rich repeats | ATTCTCTAAC | defense and stress responsiveness |
|  | TC-rich repeats | ATTCTCTAAC | defense and stress responsiveness |
|  | TATC-box | TATCCCA | gibberellin-responsiveness |
| *PtrPPO3* | TCA-element | CCATCTTTTT | salicylic acid responsiveness |
|  | ABRE | CGCACGTGTC | abscisic acid responsiveness |
|  | ABRE | ACGTG | abscisic acid responsiveness |
|  | ABRE | ACGTG | abscisic acid responsiveness |
|  | TGACG-motif | TGACG | MeJA-responsiveness |
|  | TGACG-motif | TGACG | MeJA-responsiveness |
|  | LTR | CCGAAA | low-temperature responsiveness |
|  | CGTCA-motif | CGTCA | MeJA-responsiveness |
|  | CGTCA-motif | CGTCA | MeJA-responsiveness |
| *PtrPPO4* | ABRE | CGCACGTGTC | abscisic acid responsiveness |
|  | ABRE | ACGTG | abscisic acid responsiveness |
|  | TCA-element | CCATCTTTTT | salicylic acid responsiveness |
| *PtrPPO5* | TATC-box | TATCCCA | gibberellin-responsiveness |
|  | TATC-box | TATCCCA | gibberellin-responsiveness |
|  | P-box | CCTTTTG | gibberellin-responsive element |
|  | MBS | CAACTG | drought-inducibility |
|  | TC-rich repeats | GTTTTCTTAC | defense and stress responsiveness |
|  | CGTCA-motif | CGTCA | MeJA-responsiveness |
|  | CGTCA-motif | CGTCA | MeJA-responsiveness |
|  | ABRE | ACGTG | abscisic acid responsiveness |
|  | ABRE | ACGTG | abscisic acid responsiveness |
|  | TGACG-motif | TGACG | MeJA-responsiveness |
|  | TGACG-motif | TGACG | MeJA-responsiveness |
|  | TCA-element | TCAGAAGAGG | salicylic acid responsiveness |
|  | LTR | CCGAAA | low-temperature responsiveness |
|  | RY-element | CATGCATG | seed-specific regulation |
| *PtrPPO6* | ABRE | ACGTG | abscisic acid responsiveness |
|  | TCA-element | CCATCTTTTT | salicylic acid responsiveness |
|  | MSA-like | (T/C)C(T/C)AACGG(T/C)(T/C)A | cell cycle regulation |
|  | GARE-motif | TCTGTTG | gibberellin-responsive element |
| *PtrPPO7* | CGTCA-motif | CGTCA | MeJA-responsiveness |
|  | TCA-element | CCATCTTTTT | salicylic acid responsiveness |
|  | TGACG-motif | TGACG | MeJA-responsiveness |
|  | ABRE | ACGTG | abscisic acid responsiveness |
|  | ABRE | ACGTG | abscisic acid responsiveness |
| *PtrPPO8* | P-box | CCTTTTG | gibberellin-responsive element |
|  | MBSI | aaaAaaC(G/C)GTTA | flavonoid biosynthetic genes regulation |
|  | O2-site | GATGACATGG | zein metabolism regulation |
|  | TC-rich repeats | GTTTTCTTAC | defense and stress responsiveness |
|  | CGTCA-motif | CGTCA | MeJA-responsiveness |
|  | CGTCA-motif | CGTCA | MeJA-responsiveness |
|  | TGACG-motif | TGACG | MeJA-responsiveness |
|  | TGACG-motif | TGACG | MeJA-responsiveness |
|  | ABRE | ACGTG | abscisic acid responsiveness |
|  | TCA-element | CCATCTTTTT | salicylic acid responsiveness |
|  | RY-element | CATGCATG | seed-specific regulation |
|  | LTR | CCGAAA | low-temperature responsiveness |
| *PtrPPO9* | P-box | CCTTTTG | gibberellin-responsive element |
|  | P-box | CCTTTTG | gibberellin-responsive element |
|  | TGA-element | AACGAC | auxin-responsive element |
|  | ABRE | ACGTG | abscisic acid responsiveness |
|  | ABRE | GACACGTGGC | abscisic acid responsiveness |
|  | CAT-box | GCCACT | meristem expression |
| *PtrPPO10* | GCN4_motif | TGAGTCA | endosperm expression |
|  | CAT-box | GCCACT | meristem expression |
|  | CAT-box | GCCACT | meristem expression |
|  | CGTCA-motif | CGTCA | MeJA-responsiveness |
|  | CGTCA-motif | CGTCA | MeJA-responsiveness |
|  | TGACG-motif | TGACG | MeJA-responsiveness |
|  | TGACG-motif | TGACG | MeJA-responsiveness |
|  | ABRE | ACGTG | abscisic acid responsiveness |
|  | RY-element | CATGCATG | seed-specific regulation |
|  | P-box | CCTTTTG | gibberellin-responsive element |
|  | P-box | CCTTTTG | gibberellin-responsive element |
|  | MBS | CAACTG | drought-inducibility |
|  | TC-rich repeats | GTTTTCTTAC | defense and stress responsiveness |
| *PtrPPO11* | TC-rich repeats | ATTCTCTAAC | defense and stress responsiveness |
|  | O2-site | GTTGACGTGA | zein metabolism regulation |
|  | TCA-element | CCATCTTTTT | salicylic acid responsiveness |
|  | TGACG-motif | TGACG | MeJA-responsiveness |
|  | TGACG-motif | TGACG | MeJA-responsiveness |
|  | ABRE | ACGTG | abscisic acid responsiveness |
|  | ABRE | AACCCGG | abscisic acid responsiveness |
|  | ABRE | GCCGCGTGGC | abscisic acid responsiveness |
|  | CGTCA-motif | CGTCA | MeJA-responsiveness |
|  | CGTCA-motif | CGTCA | MeJA-responsiveness |
| *PtrPPO12* | TCA-element | CCATCTTTTT | salicylic acid responsiveness |
|  | ABRE | ACGTG | abscisic acid responsiveness |
|  | ABRE | ACGTG | abscisic acid responsiveness |
|  | ABRE | ACGTG | abscisic acid responsiveness |
|  | LTR | CCGAAA | low-temperature responsiveness |
| *PtrPPO13* | TCA-element | CCATCTTTTT | salicylic acid responsiveness |
|  | ABRE | AACCCGG | abscisic acid responsiveness |
|  | ABRE | CACGTG | abscisic acid responsiveness |
|  | ABRE | ACGTG | abscisic acid responsiveness |
|  | ABRE | ACGTG | abscisic acid responsiveness |
|  | TGACG-motif | TGACG | MeJA-responsiveness |
|  | CAT-box | GCCACT | meristem expression |
|  | CAT-box | GCCACT | meristem expression |
|  | CGTCA-motif | CGTCA | MeJA-responsiveness |
|  | TGA-element | AACGAC | auxin-responsive element |
|  | P-box | CCTTTTG | gibberellin-responsive element |
|  | P-box | CCTTTTG | gibberellin-responsive element |
|  | TC-rich repeats | GTTTTCTTAC | defense and stress responsiveness |
|  | TC-rich repeats | GTTTTCTTAC | defense and stress responsiveness |
|  | GC-motif | CCCCCG | anoxic specific inducibility |
| *PtrPPO14* | TGACG-motif | TGACG | MeJA-responsiveness |
|  | TGACG-motif | TGACG | MeJA-responsiveness |
|  | TGACG-motif | TGACG | MeJA-responsiveness |
|  | CGTCA-motif | CGTCA | MeJA-responsiveness |
|  | CGTCA-motif | CGTCA | MeJA-responsiveness |
|  | CGTCA-motif | CGTCA | MeJA-responsiveness |
|  | GCN4_motif | TGAGTCA | endosperm expression |
|  | CAT-box | GCCACT | meristem expression |
|  | O2-site | GATGACATGG | zein metabolism regulation |
|  | MBS | CAACTG | drought-inducibility |
|  | MBS | CAACTG | drought-inducibility |
|  | P-box | CCTTTTG | gibberellin-responsive element |
| *PtrPPO15* | O2-site | GTTGACGTGA | zein metabolism regulation |
|  | O2-site | GATGA(C/T)(A/G)TG(A/G) | zein metabolism regulation |
|  | CGTCA-motif | CGTCA | MeJA-responsiveness |
|  | CGTCA-motif | CGTCA | MeJA-responsiveness |
|  | HD-Zip 1 | CAAT(A/T)ATTG | palisade mesophyll cells differentiation |
|  | ABRE | ACGTG | abscisic acid responsiveness |
|  | ABRE | ACGTG | abscisic acid responsiveness |
|  | TGACG-motif | TGACG | MeJA-responsiveness |
|  | TGACG-motif | TGACG | MeJA-responsiveness |
| *PtrPPO16* | O2-site | GATGACATGG | zein metabolism regulation |
|  | TC-rich repeats | GTTTTCTTAC | defense and stress responsiveness |
|  | P-box | CCTTTTG | gibberellin-responsive element |
|  | LTR | CCGAAA | low-temperature responsiveness |
|  | ABRE | ACGTG | abscisic acid responsiveness |
|  | TGACG-motif | TGACG | MeJA-responsiveness |
|  | TGACG-motif | TGACG | MeJA-responsiveness |
|  | TCA-element | CCATCTTTTT | salicylic acid responsiveness |
|  | CGTCA-motif | CGTCA | MeJA-responsiveness |
|  | CGTCA-motif | CGTCA | MeJA-responsiveness |
| *PtrPPO17* | CGTCA-motif | CGTCA | MeJA-responsiveness |
|  | ABRE | ACGTG | abscisic acid responsiveness |
|  | ABRE | ACGTG | abscisic acid responsiveness |
|  | TGACG-motif | TGACG | MeJA-responsiveness |
|  | TCA-element | CCATCTTTTT | salicylic acid responsiveness |
| *PtrPPO18* | ABRE | ACGTG | abscisic acid responsiveness |
|  | TCA-element | CCATCTTTTT | salicylic acid responsiveness |
|  | CAT-box | GCCACT | meristem expression |

**Table S7. The number of functional elements in PPOs gene**

| **Gene ID** | **MeJA-responsiveness** | **salicylic acid responsiveness** | **abscisic acid responsiveness** | **drought-inducibility** | **defense and stress responsiveness** | **low-temperature responsiveness** | **zein metabolism regulation** | **flavonoid biosynthetic genes regulation** | **gibberellin responsiveness** | **auxin-responsive** | **cell cycle regulation** | **meristem expression** | **seed-specific regulation** | **anoxic specific inducibility** | **endosperm expression** | **palisade mesophyll cells differentiation** |
| --- | --- | --- | --- | --- | --- | --- | --- | --- | --- | --- | --- | --- | --- | --- | --- | --- |
| *PtrPPO1* | 2 | 1 | 1 | / | 1 | / | 1 | / | 1 | / | / | / | / | / | / | / |
| *PtrPPO2* | 2 | / | 1 | 2 | 2 | / | / | / | 1 | / | / | / | / | / | / | / |
| *PtrPPO3* | 4 | 1 | 3 | / | / | 1 | / | / | / | / | / | / | / | / | / | / |
| *PtrPPO4* | / | 1 | 2 | / | / | / | / | / | / | / | / | / | / | / | / | / |
| *PtrPPO5* | 4 | 1 | 2 | 1 | 1 | 1 | / | / | 3 | / | / | / | 1 | / | / | / |
| *PtrPPO6* | / | 1 | 1 | / | / | / | / | / | 1 | / | 1 | / | / | / | / | / |
| *PtrPPO7* | 2 | 1 | 2 | / | / | / | / | / | / | / | / | / | / | / | / | / |
| *PtrPPO8* | 4 | 1 | 1 | / | 1 | 1 | 1 | 1 | 1 | / | / | / | 1 | / | / | / |
| *PtrPPO9* | / | / | 2 | / | / | / | / | / | 2 | 1 | / | 1 | / | / | / | / |
| *PtrPPO10* | 4 | / | 1 | 1 | 1 | / | / | / | 2 | / | 1 | 2 | / | / | 1 | / |
| *PtrPPO11* | 4 | 1 | 3 | / | 1 | / | 1 | / | / | / | / | / | / | / | / | / |
| *PtrPPO12* | / | 1 | 3 | / | / | 1 | / | / | / | / | / | / | / | / | / | / |
| *PtrPPO13* | 2 | 1 | 4 | / | 2 | / | / | / | 2 | 1 | / | 2 | / | 1 | / | / |
| *PtrPPO14* | 6 | / | / | 2 | / | / | 1 | / | 1 | / | / | 1 | / | / | 1 | / |
| *PtrPPO15* | 4 | / | 2 | / | / | / | 2 | / | / | / | / | / | / | / | / | 1 |
| *PtrPPO16* | 4 | 1 | 1 | / | 1 | 1 | 1 | / | 1 | / | / | / | / | / | / | / |
| *PtrPPO17* | 2 | 1 | 2 | / | / | / | / | / | / | / | / | / | / | / | / | / |
| *PtrPPO18* | / | 1 | 1 | / | / | / | / | / | / | / | / | 1 | / | / | / | / |

**Table S8. Expression of PPOs gene in different plant tissues**

| **GeneID** | **Twigs-Non-Girdled** | **Flowers-Dormant** | **Flowers-Expanded** | **Leaves-Mature** | **Flowers-Expanding** | **Suckers-Whole-Sucker** | **Petiole-Mature** | **Buds-Prechilling** | **Buds-Dormant** | **Leaves-Freshly-Expanded** | **Leaves-Non-Girdled** | **Leaves-Girdled** | **Seeds-Mature** | **Leaves-Young-Expanding** | **Cambium-Phloem-Dormant** |
| --- | --- | --- | --- | --- | --- | --- | --- | --- | --- | --- | --- | --- | --- | --- | --- |
| ***PtrPPO1*** | -0.77 | 1.29 | 0.29 | -0.12 | 0.37 | 0.75 | -0.69 | -0.68 | -0.46 | 2.00 | 0.18 | 0.12 | 0.85 | 2.14 | -0.96 |
| ***PtrPPO2*** | 0.00 | 0.00 | 0.00 | 0.00 | 0.00 | 0.00 | 0.00 | 0.00 | 0.00 | 0.00 | 0.00 | 0.00 | 0.00 | 0.00 | 0.00 |
| ***PtrPPO3*** | 0.88 | 2.85 | 0.85 | -0.99 | -0.15 | 1.12 | -0.99 | 3.83 | 4.17 | 1.53 | 0.36 | -0.99 | 0.36 | 3.81 | -0.99 |
| ***PtrPPO4*** | -0.63 | 1.66 | -1.45 | -1.60 | -0.18 | 1.53 | -1.16 | 2.51 | 2.83 | 1.90 | -0.73 | -1.73 | 0.54 | 3.69 | -2.24 |
| ***PtrPPO5*** | 0.18 | 1.97 | -1.08 | -1.75 | -0.03 | 1.38 | -1.13 | 2.97 | 3.20 | 1.88 | -0.54 | -0.87 | 0.66 | 3.80 | -2.04 |
| ***PtrPPO6*** | -0.08 | 1.14 | -0.89 | 0.05 | 0.91 | 0.24 | -0.08 | 0.53 | 1.51 | 0.93 | -0.89 | 0.71 | 1.15 | 2.10 | -0.89 |
| ***PtrPPO7*** | 1.56 | 1.88 | 0.00 | 0.00 | 1.18 | 1.12 | 0.00 | 3.13 | 3.40 | 0.00 | 0.00 | 0.00 | 0.00 | 2.81 | 0.00 |
| ***PtrPPO8*** | 0.06 | -0.75 | -0.75 | -0.75 | 0.43 | 0.37 | -0.75 | 0.96 | 0.18 | -0.75 | -0.06 | 0.58 | -0.75 | 1.45 | -0.75 |
| ***PtrPPO9*** | -1.23 | 0.00 | 1.79 | -3.52 | 0.63 | 0.97 | -3.10 | 0.48 | -1.84 | 0.92 | -3.74 | -2.04 | 3.18 | 0.00 | -6.44 |
| ***PtrPPO10*** | 0.08 | -1.16 | 0.89 | 0.33 | -0.08 | -0.37 | -0.08 | -0.29 | -1.73 | -1.25 | -0.31 | 0.57 | 0.72 | -2.04 | -0.89 |
| ***PtrPPO11*** | -1.68 | 2.09 | 0.40 | -0.49 | 1.29 | 1.61 | -2.10 | 1.49 | 1.72 | 2.31 | 1.33 | 0.65 | 0.27 | 0.09 | -3.19 |
| ***PtrPPO12*** | -1.65 | -0.55 | 0.74 | 1.45 | -0.69 | -0.12 | -0.38 | 0.16 | -1.82 | -1.64 | 1.65 | 0.12 | 1.24 | -3.20 | -4.65 |
| ***PtrPPO13*** | 0.88 | 2.50 | 1.82 | -1.60 | 0.70 | 0.64 | -0.13 | 2.39 | -2.62 | -1.89 | -0.08 | -0.95 | 2.23 | -3.82 | -6.39 |
| ***PtrPPO14*** | 0.04 | 0.34 | -1.83 | -0.52 | 0.25 | 4.70 | -1.03 | -1.83 | -0.27 | 6.26 | -1.14 | -0.88 | -0.48 | 4.68 | -0.59 |
| ***PtrPPO15*** | 0.26 | 1.27 | -1.51 | -1.04 | 1.11 | 5.62 | -2.62 | -1.19 | -0.49 | 7.15 | -1.43 | -0.79 | -0.26 | 5.43 | -0.72 |
| ***PtrPPO16*** | 0.01 | 1.06 | -0.01 | -1.55 | 0.08 | 1.11 | -1.55 | 1.39 | 1.44 | 1.26 | -0.57 | -1.55 | -1.55 | 2.57 | -1.55 |
| ***PtrPPO17*** | 0.00 | 0.89 | 0.00 | 0.00 | 0.00 | 0.80 | 0.00 | 1.02 | 1.30 | 0.00 | 0.00 | 0.00 | 0.00 | 1.49 | 0.00 |
| ***PtrPPO18*** | 1.02 | 2.85 | -0.55 | -2.09 | -0.46 | 1.82 | -2.09 | 4.37 | 4.33 | 2.32 | -1.39 | -1.14 | -0.05 | 5.39 | -2.09 |

| **GeneID** | **Leaves-Beetle-**  **Damaged** | **Leaves-Mechanical-**  **Damage** | **Leaves-**  **Drought** | **Roots-**  **Drought** |
| --- | --- | --- | --- | --- |
| *PtrPPO1* | 1.27 | 1.23 | -0.61 | -1.25 |
| *PtrPPO2* | 0.00 | 0.00 | 0.00 | 0.00 |
| *PtrPPO3* | -0.08 | 0.94 | -0.05 | -0.85 |
| *PtrPPO4* | 0.05 | 0.51 | 0.01 | -1.56 |
| *PtrPPO5* | -0.39 | 0.22 | -0.30 | -1.35 |
| *PtrPPO6* | 0.84 | 1.22 | 0.00 | 0.00 |
| *PtrPPO7* | -0.91 | 0.56 | -0.91 | 0.00 |
| *PtrPPO8* | 0.84 | 1.22 | 0.00 | -1.44 |
| *PtrPPO9* | -0.09 | 0.30 | 4.32 | 0.02 |
| *PtrPPO10* | -0.07 | 0.27 | -0.17 | 0.30 |
| *PtrPPO11* | -2.27 | -1.86 | -0.65 | -0.61 |
| *PtrPPO12* | 0.81 | 0.57 | -0.29 | -1.25 |
| *PtrPPO13* | 3.15 | 3.68 | 4.11 | 1.06 |
| *PtrPPO14* | -3.14 | -2.99 | -1.26 | -0.66 |
| *PtrPPO15* | -3.19 | -3.26 | -1.47 | -1.96 |
| *PtrPPO16* | 0.08 | 0.73 | 1.12 | -0.85 |
| *PtrPPO17* | 0.84 | 0.00 | 0.00 | 0.00 |
| *PtrPPO18* | 0.83 | 1.24 | 0.50 | -1.64 |

**Table S9. Expression of PPOs gene in different treatments**

**Table S10. Potential upstream transcription factors of *PtrPPOs***

| **TF** | **Description** | **Target** | **Method** | **Position** | **Strand** | **Sequence** | **P-value** |
| --- | --- | --- | --- | --- | --- | --- | --- |
| Potri.002G026700 | BBR-BPC family protein | PtrPPO9 | motif | Chr01:40682931-40682951 | - | ACACTTTTTTCTCTCTCTCGA | 0.00000438 |
| Potri.002G151700 | MIKC_MADS family protein | PtrPPO9 | motif | Chr01:40682926-40682946 | - | TTTTTCTCTCTCTCGATTCTC | 3.71E-06 |
| Potri.003G034200 | Dof family protein | PtrPPO9 | motif | Chr01:40682881-40682901 | + | AAGCACGAAAAGTAAATAAGA | 2.13E-06 |
| Potri.003G207200 | bHLH family protein | PtrPPO9 | motif | Chr01:40683022-40683030 | + | GCCACTTGT | 0.00000736 |
| Potri.004G056900 | Dof family protein | PtrPPO9 | motif | Chr01:40682934-40682954 | + | AGAGAGAGAAAAAAGTGTACG | 1.39E-06 |
| Potri.006G132400 | AP2 family protein | PtrPPO9 | motif | Chr01:40682814-40682823 | - | TCCTCGGACA | 4.92E-06 |
| Potri.006G224100 | WRKY family protein | PtrPPO9 | motif | Chr01:40683002-40683014 | - | ATTTGACTTTTCT | 7.13E-06 |
| Potri.009G064700 | bHLH family protein | PtrPPO9 | motif | Chr01:40683022-40683030 | + | GCCACTTGT | 7.36E-06 |
| Potri.010G181000 | AP2 family protein | PtrPPO9 | motif | Chr01:40682824-40682843 | - | CTAAAAGAAAAAAAAAGAGG | 0.00000492 |
| Potri.014G074200 | MIKC_MADS family protein | PtrPPO9 | motif | Chr01:40682926-40682946 | - | TTTTTCTCTCTCTCGATTCTC | 3.71E-06 |
| Potri.001G238400 | Dof family protein | PtrPPO13 | motif | Chr11:4046510-4046530,Chr11:4046503-4046523,Chr11:4046496-4046516 | +,+,+ | CTTTTTTCTTTTTTCTTTTTC,CTTTTTTCTTTTTTCTTTTTT,TTTTTTTCTTTTTTCTTTTTT | 8.28e-08,2.41e-07,1. |
| Potri.001G322700 | B3 family protein | PtrPPO13 | motif | Chr11:4046956-4046970 | + | AGATGCATGCATCGG | 6.68E-06 |
| Potri.002G026700 | BBR-BPC family protein | PtrPPO13 | motif | Chr11:4046972-4046992,Chr11:4046970-4046990,Chr11:4046968-4046988,Chr11:4046966-4046986,Chr11:404696 | +,+,+,+,+, | CTCTCTCTCTCTCTCTTGTAT,GTCTCTCTCTCTCTCTCTTGT,CGGTCTCTCTCTCTCTCTCTT,ATCGGTCTCTCTCTCTCTCTC,GCATCGGTCTCT | 4.44e-06,1.83e-08,1. |
| Potri.002G113300 | TALE family protein | PtrPPO13 | motif | Chr11:4046970-4046989,Chr11:4046968-4046987 | +,+ | GTCTCTCTCTCTCTCTCTTG,CGGTCTCTCTCTCTCTCTCT | 3.04e-06,1.08e-07 |
| Potri.002G151700 | MIKC_MADS family protein | PtrPPO13 | motif | Chr11:4046969-4046989,Chr11:4046548-4046568,Chr11:4046547-4046567,Chr11:4046546-4046566,Chr11:404654 | +,-,-,-,-, | GGTCTCTCTCTCTCTCTCTTG,TCTTCGTTTTTTTTTTTTTTT,CTTCGTTTTTTTTTTTTTTTT,TTCGTTTTTTTTTTTTTTTTT,CGTTTTTTTTTT | 7.82e-08,1e-06,1.32e |
| Potri.002G188700 | SBP family protein | PtrPPO13 | motif | Chr11:4046719-4046727 | + | TGGTACGGC | 8.25E-06 |
| Potri.002G252000 | B3 family protein | PtrPPO13 | motif | Chr11:4046724-4046732 | + | CGGCATGCA | 3.66E-06 |
| Potri.003G034200 | Dof family protein | PtrPPO13 | motif | Chr11:4046515-4046535,Chr11:4046508-4046528,Chr11:4046501-4046521,Chr11:4046494-4046514 | -,-,-,- | TGCAAGAAAAAGAAAAAAGAA,AAAAGAAAAAAGAAAAAAGAA,AAAAGAAAAAAGAAAAAAGAA,AAAAGAAAAAAGAAAAAAAAG | 3.84e-06,2.92e-07,2. |
| Potri.004G056900 | Dof family protein | PtrPPO13 | motif | Chr11:4046511-4046531,Chr11:4046504-4046524,Chr11:4046497-4046517 | -,-,- | AGAAAAAGAAAAAAGAAAAAA,GAAAAAAGAAAAAAGAAAAAA,GAAAAAAGAAAAAAGAAAAAA | 1.68e-06,1.22e-06,1. |
| Potri.007G011600 | AP2 family protein | PtrPPO13 | motif | Chr11:4046690-4046704 | - | CCTCGGAATCTGGTC | 5.76E-06 |
| Potri.007G023600 | bHLH family protein | PtrPPO13 | motif | Chr11:4046818-4046829 | + | CGCGCATGTGCA | 6.87E-06 |
| Potri.007G036400 | Dof family protein | PtrPPO13 | motif | Chr11:4046509-4046529,Chr11:4046502-4046522,Chr11:4046495-4046515 | +,+,+ | TCTTTTTTCTTTTTTCTTTTT,TCTTTTTTCTTTTTTCTTTTT,TTTTTTTTCTTTTTTCTTTTT | 3.29e-07,3.29e-07,5. |
| Potri.008G131700 | GRAS family protein | PtrPPO13 | motif | Chr11:4046969-4046988,Chr11:4046967-4046986,Chr11:4046513-4046532 | -,-,- | AAGAGAGAGAGAGAGAGACC,GAGAGAGAGAGAGAGACCGA,AAGAAAAAGAAAAAAGAAAA | 3.6e-06,2.73e-07,1.2 |
| Potri.010G101400 | BBR-BPC family protein | PtrPPO13 | motif | Chr11:4046969-4046992,Chr11:4046967-4046990,Chr11:4046965-4046988,Chr11:4046963-4046986,Chr11:404650 | -,-,-,-,- | ATACAAGAGAGAGAGAGAGAGACC,ACAAGAGAGAGAGAGAGAGACCGA,AAGAGAGAGAGAGAGAGACCGATG,GAGAGAGAGAGAGAGACCGATGCA, | 7.64e-06,1.06e-08,2. |
| Potri.010G181000 | AP2 family protein | PtrPPO13 | motif | Chr11:4046973-4046992,Chr11:4046971-4046990,Chr11:4046969-4046988,Chr11:4046548-4046567,Chr11:404654 | -,-,-,+,+, | ATACAAGAGAGAGAGAGAGA,ACAAGAGAGAGAGAGAGAGA,AAGAGAGAGAGAGAGAGACC,AAAAAAAAAAAAAAACGAAG,AAAAAAAAAAAAAAAA | 6.86e-06,7.33e-07,4. |
| Potri.010G204800 | Dof family protein | PtrPPO13 | motif | Chr11:4046546-4046566,Chr11:4046543-4046563,Chr11:4046542-4046562,Chr11:4046541-4046561,Chr11:404654 | -,-,-,-,-, | TTCGTTTTTTTTTTTTTTTTT,GTTTTTTTTTTTTTTTTTTTT,TTTTTTTTTTTTTTTTTTTTT,TTTTTTTTTTTTTTTTTTTTT,TTTTTTTTTTTT | 5.5e-06,4.98e-06,2.1 |
| Potri.012G081300 | Dof family protein | PtrPPO13 | motif | Chr11:4046518-4046538,Chr11:4046511-4046531,Chr11:4046504-4046524,Chr11:4046497-4046517 | -,-,-,- | GGATGCAAGAAAAAGAAAAAA,AGAAAAAGAAAAAAGAAAAAA,GAAAAAAGAAAAAAGAAAAAA,GAAAAAAGAAAAAAGAAAAAA | 6.23e-06,2.76e-07,1. |
| Potri.014G074200 | MIKC_MADS family protein | PtrPPO13 | motif | Chr11:4046969-4046989,Chr11:4046548-4046568,Chr11:4046547-4046567,Chr11:4046546-4046566,Chr11:404654 | +,-,-,-,-, | GGTCTCTCTCTCTCTCTCTTG,TCTTCGTTTTTTTTTTTTTTT,CTTCGTTTTTTTTTTTTTTTT,TTCGTTTTTTTTTTTTTTTTT,CGTTTTTTTTTT | 7.82e-08,1e-06,1.32e |
| Potri.019G081100 | C2H2 family protein | PtrPPO13 | motif | Chr11:4046397-4046415 | - | CTTCTATCTCTCCTTCCAC | 6.40E-06 |
| Potri.001G238400 | Dof family protein | PtrPPO11 | motif | Chr04:17828950-17828970 | + | ATCAATTTTTTTTGCTTTTTT | 5.44E-06 |
| Potri.003G121200 | ERF family protein | PtrPPO11 | motif | Chr04:17829169-17829183 | + | TTTTGTCGGCATAAC | 9.26E-06 |
| Potri.004G078200 | ARF family protein | PtrPPO11 | motif | Chr04:17829170-17829178 | + | TTTGTCGGC | 6.50E-06 |
| Potri.005G236700 | ARF family protein | PtrPPO11 | motif | Chr04:17829169-17829178 | - | GCCGACAAAA | 9.88E-07 |
| Potri.006G084200 | Dof family protein | PtrPPO11 | motif | Chr04:17828956-17828970 | + | TTTTTTTGCTTTTTT | 6.51E-06 |
| Potri.007G036400 | Dof family protein | PtrPPO11 | motif | Chr04:17828949-17828969 | + | TATCAATTTTTTTTGCTTTTT | 7.57E-06 |
| Potri.012G106100 | ARF family protein | PtrPPO11 | motif | Chr04:17829170-17829179 | - | TGCCGACAAA | 6.24E-06 |
| Potri.001G053500 | GATA family protein | PtrPPO14 | motif | Chr11:13227931-13227945 | + | GTGGTAGTGTTGGAG | 5.76E-06 |
| Potri.001G163700 | ERF family protein | PtrPPO14 | motif | Chr11:13228204-13228224 | + | GGGTTTGTATGACGGCTGCTG | 4.64E-06 |
| Potri.001G356100 | ERF family protein | PtrPPO14 | motif | Chr11:13228213-13228227 | + | TGACGGCTGCTGTTA | 8.94E-06 |
| Potri.002G009700 | Nin-like family protein | PtrPPO14 | motif | Chr11:13228176-13228190,Chr11:13228179-13228193 | -,- | GCAGCAGCAACCATC,TTCGCAGCAGCAACC | 1.88e-06,9.98e-07 |
| Potri.002G026700 | BBR-BPC family protein | PtrPPO14 | motif | Chr11:13228015-13228035,Chr11:13228019-13228039 | -,- | TTCTCCTTCTTTCTCTCATCA,TTCTTTCTCCTTCTTTCTCTC | 3.86e-06,5.81e-08 |
| Potri.002G151700 | MIKC_MADS family protein | PtrPPO14 | motif | Chr11:13228016-13228036,Chr11:13228020-13228040 | -,- | TTTCTCCTTCTTTCTCTCATC,GTTCTTTCTCCTTCTTTCTCT | 8.07e-06,5.32e-07 |
| Potri.005G164900 | MYB family protein | PtrPPO14 | motif | Chr11:13227964-13227984 | + | TGATTAGTGTTTGTTGAACAA | 9.65E-06 |
| Potri.008G079800 | LBD family protein | PtrPPO14 | motif | Chr11:13228216-13228236 | - | TCGCTTCCATAACAGCAGCCG | 6.42E-06 |
| Potri.008G131700 | GRAS family protein | PtrPPO14 | motif | Chr11:13228023-13228042 | + | GAAAGAAGGAGAAAGAACAG | 7.02E-06 |
| Potri.010G101400 | BBR-BPC family protein | PtrPPO14 | motif | Chr11:13228015-13228038,Chr11:13228017-13228040,Chr11:13228019-13228042,Chr11:13228021-13228044 | +,+,+,+ | TGATGAGAGAAAGAAGGAGAAAGA,ATGAGAGAAAGAAGGAGAAAGAAC,GAGAGAAAGAAGGAGAAAGAACAG,GAGAAAGAAGGAGAAAGAACAGAG | 1.31e-06,1.15e-07,9. |
| Potri.010G177100 | LBD family protein | PtrPPO14 | motif | Chr11:13228216-13228236 | - | TCGCTTCCATAACAGCAGCCG | 6.42E-06 |
| Potri.010G181000 | AP2 family protein | PtrPPO14 | motif | Chr11:13228017-13228036,Chr11:13228019-13228038,Chr11:13228021-13228040,Chr11:13228061-13228080,Chr1 | +,+,+,+,+ | ATGAGAGAAAGAAGGAGAAA,GAGAGAAAGAAGGAGAAAGA,GAGAAAGAAGGAGAAAGAAC,ACAACCAAAAAAAAAAGAGA,AAAAAAAAGAGAGCAA | 8.05e-06,5.82e-07,6. |
| Potri.012G083500 | LBD family protein | PtrPPO14 | motif | Chr11:13228214-13228234 | - | GCTTCCATAACAGCAGCCGTC | 4.79E-06 |
| Potri.013G101100 | ERF family protein | PtrPPO14 | motif | Chr11:13228175-13228194 | - | CTTCGCAGCAGCAACCATCA | 4.92E-06 |
| Potri.014G074200 | MIKC_MADS family protein | PtrPPO14 | motif | Chr11:13228016-13228036,Chr11:13228020-13228040 | -,- | TTTCTCCTTCTTTCTCTCATC,GTTCTTTCTCCTTCTTTCTCT | 8.07e-06,5.32e-07 |
| Potri.015G023200 | ERF family protein | PtrPPO14 | motif | Chr11:13228204-13228224 | - | CAGCAGCCGTCATACAAACCC | 7.41E-06 |
| Potri.015G082200 | LBD family protein | PtrPPO14 | motif | Chr11:13228214-13228234 | - | GCTTCCATAACAGCAGCCGTC | 4.79E-06 |
| Potri.015G089000 | C2H2 family protein | PtrPPO14 | motif | Chr11:13228035-13228046 | + | AAGAACAGAGTA | 4.56E-06 |
| Potri.017G013700 | ERF family protein | PtrPPO14 | motif | Chr11:13228175-13228195,Chr11:13228204-13228224 | +,+ | TGATGGTTGCTGCTGCGAAGG,GGGTTTGTATGACGGCTGCTG | 6.33e-06,6.33e-06 |
| Potri.018G028000 | ERF family protein | PtrPPO14 | motif | Chr11:13228176-13228196 | - | CCCTTCGCAGCAGCAACCATC | 8.00E-06 |
| Potri.010G101400 | BBR-BPC family protein | PtrPPO15 | motif | Chr11:13259196-13259219 | - | GTGAGGCTGAAAGTAGGAGAGAAA | 2.43E-06 |

**Table S11. Expression of potential upstream transcription factors in different treatments**

| **Target** | **TFs** | **Roots-Drought** | **Leaves-Drought** | **Leaves-Beetle-Damaged** | **Leaves-Mechanical-Damage** |
| --- | --- | --- | --- | --- | --- |
| PtrPPO9 | Potri.002G026700(BBR-BPC) | -0.167491983 | -0.190816858 | -0.356840702 | -0.352310009 |
|  | Potri.002G151700(MIKC_MADS) | -1.212651903 | 1.059969249 | 0.515943238 | 1.010145734 |
|  | Potri.003G034200(Dof) | 0.501687761 | -0.353385379 | 1.071208144 | -0.056045355 |
|  | Potri.003G207200(bHLH) | -0.10412806 | 1.591625447 | 1.100959527 | 1.373980686 |
|  | Potri.004G056900(Dof) | -0.600052071 | -0.346378165 | 0.988839889 | 0.056260532 |
|  | Potri.006G132400(AP2) | -0.241706526 | 0.266893876 | -0.243466643 | 0.265037466 |
|  | Potri.006G224100(WRKY) | -0.598060951 | -0.120812313 | 0.302020808 | -0.015041925 |
|  | Potri.009G064700(bHLH) | 0.062138839 | -0.578905774 | 1.289990613 | 0.330486407 |
|  | Potri.010G181000(AP2) | 0.637035279 | 0 | 0 | 0 |
|  | Potri.014G074200(MIKC_MADS) | -0.380911308 | 0.882979059 | -1.490497126 | -0.318166906 |
| PtrPPO11 | Potri.001G238400(Dof) | -0.449565132 | 1.009304636 | -0.750792697 | -0.282205926 |
|  | Potri.003G121200(ERF) | 1.06272895 | 0 | 0 | 0 |
|  | Potri.004G078200(ARF) | 0.090869548 | -0.196367863 | 0.445650456 | -0.008349071 |
|  | Potri.005G236700(ARF) | 0.067837052 | 0.318002665 | 2.571839798 | 2.251425792 |
|  | Potri.006G084200(Dof) | 0.023339616 | 0.060353551 | -0.069660727 | -0.530644332 |
|  | Potri.007G036400(Dof) | -0.036651463 | 0.914753346 | -2.087952852 | -1.214307866 |
|  | Potri.012G106100(ARF) | 0.430735912 | -0.273417551 | 0.999139071 | 0.548985457 |
| PtrPPO13 | Potri.001G238400(Dof) | -0.449565132 | 1.009304636 | -0.750792697 | -0.282205926 |
|  | Potri.001G322700(B3) | -0.289579761 | 0.649270654 | -0.913433662 | -0.913433662 |
|  | Potri.002G026700(BBR-BPC) | -0.167491983 | -0.190816858 | -0.356840702 | -0.352310009 |
|  | Potri.002G113300(TALE) | -0.69222323 | -1.278497844 | -2.087952852 | -2.087952852 |
|  | Potri.002G151700(MIKC_MADS) | -1.212651903 | 1.059969249 | 0.515943238 | 1.010145734 |
|  | Potri.002G188700(SBP) | -0.026625528 | -0.27294538 | 0.091928612 | -0.156247145 |
|  | Potri.002G252000(B3) | 0 | 0 | 0 | 0 |
|  | Potri.003G034200(Dof) | 0.501687761 | -0.353385379 | 1.071208144 | -0.056045355 |
|  | Potri.004G056900(Dof) | -0.600052071 | -0.346378165 | 0.988839889 | 0.056260532 |
|  | Potri.007G011600(AP2) | -0.543539999 | 0 | 0 | 0.873644986 |
|  | Potri.007G023600(bHLH) | 0.227148262 | -0.456383783 | -2.504449468 | -1.76776136 |
|  | Potri.007G036400(Dof) | -0.036651463 | 0.914753346 | -2.087952852 | -1.214307866 |
|  | Potri.008G131700(GRAS) | -0.227072213 | 0.335305128 | 0.941390691 | 0.619208827 |
|  | Potri.010G101400(BBR-BPC) | 0.190462099 | 0.245480602 | -0.174179748 | -0.461848717 |
|  | Potri.010G181000(AP2) | 0.637035279 | 0 | 0 | 0 |
|  | Potri.010G204800(Dof) | 0 | 0 | 0 | 0 |
|  | Potri.012G081300(Dof) | -0.621747583 | 0.861256702 | 0.005511478 | 0.111843811 |
|  | Potri.014G074200(MIKC_MADS) | -0.380911308 | 0.882979059 | -1.490497126 | -0.318166906 |
|  | Potri.019G081100(C2H2) | -0.395597155 | 0.087125676 | 0.976854343 | 0.309465775 |
| PtrPPO14 | Potri.001G053500(GATA) | 0.232800669 | 0.206866659 | 0.083359639 | -0.063655096 |
|  | Potri.001G163700(ERF) | 0.26339612 | 0.491058318 | 0.440319429 | 0.584705991 |
|  | Potri.001G356100(ERF) | 0.682018672 | 0.536969785 | -0.717146795 | -0.020138825 |
|  | Potri.002G009700(Nin-like) | -0.034065087 | 0.082606571 | 0.531390182 | 0.817573022 |
|  | Potri.002G026700(BBR-BPC) | -0.167491983 | -0.190816858 | -0.356840702 | -0.352310009 |
|  | Potri.002G151700(MIKC_MADS) | -1.212651903 | 1.059969249 | 0.515943238 | 1.010145734 |
|  | Potri.005G164900(MYB) | -0.464846225 | 0 | 0 | 0.873644986 |
|  | Potri.008G079800(LBD) | 0.77731274 | 0 | 0 | 0 |
|  | Potri.008G131700(GRAS) | -0.227072213 | 0.335305128 | 0.941390691 | 0.619208827 |
|  | Potri.010G101400(BBR-BPC) | 0.190462099 | 0.245480602 | -0.174179748 | -0.461848717 |
|  | Potri.010G177100(LBD) | -1.189435601 | 1.869507274 | -2.228904985 | -1.355259999 |
|  | Potri.010G181000(AP2) | 0.637035279 | 0 | 0 | 0 |
|  | Potri.012G083500(LBD) | -0.962179115 | 0.455149502 | -0.913433662 | -0.039788677 |
|  | Potri.013G101100(ERF) | 2.666537257 | 0 | 1.412801442 | 0 |
|  | Potri.014G074200(MIKC_MADS) | -0.380911308 | 0.882979059 | -1.490497126 | -0.318166906 |
|  | Potri.015G023200(ERF) | 0.122148305 | 0.584221413 | 0.789966834 | 0.039174379 |
|  | Potri.015G082200(LBD) | 0.115802573 | 1.511041194 | -0.076549736 | 0.30455631 |
|  | Potri.015G089000(C2H2) | 0.371394785 | -0.59458127 | -0.46627485 | -0.807080025 |
|  | Potri.017G013700(ERF) | 1.888683409 | 1.964394844 | 0.667612158 | -0.037129611 |
|  | Potri.018G028000(ERF) | -1.079062402 | -2.063768504 | -6.810352181 | -6.810352181 |
| PtrPPO15 | Potri.010G101400(BBR-BPC) | 0.190462099 | 0.245480602 | -0.174179748 | -0.461848717 |


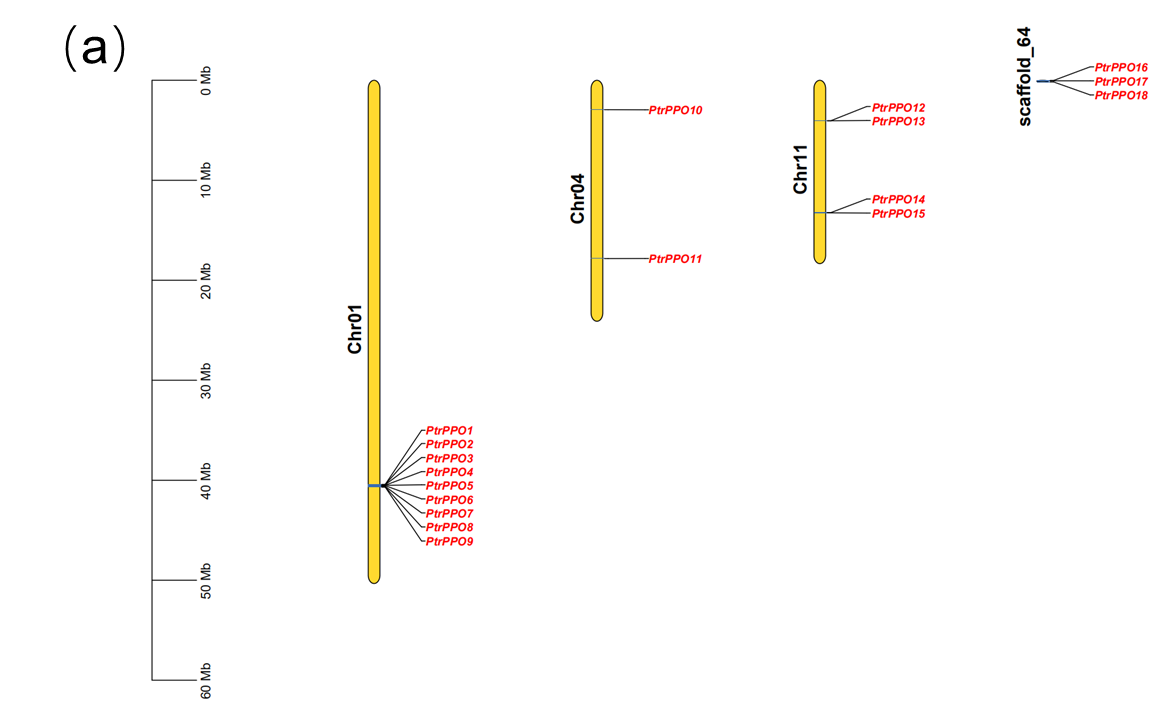


Figure S1 Chromosomal locations of Populus PPO family members. The chromosomal locations of the PPO genes were mapped with MapDraw program.
